# Iron release dynamics in Indo-Gangetic alluvial soils under variable moisture conditions: Insights from multi-model kinetic and geochemical speciation analysis

**DOI:** 10.1371/journal.pone.0355846

**Published:** 2026-08-28

**Authors:** Debrup Ghosh, Mandira Barman, Karnena Koteswara Rao, Debarup Das, Siba Prasad Datta, Manoj Shrivastava, Hari Krishna, Debashis Mandal

**Affiliations:** 1 ICAR-Indian Agricultural Research Institute, New Delhi, India; 2 Regional Coastal Rice Research Station, ICAR-Central Rice Research Institute, Naira, Andhra Pradesh, India; 3 Indian Institute of Soil Science, Bhopal, India; IIT Roorkee: Indian Institute of Technology Roorkee, INDIA

## Abstract

Iron (Fe) plays a significant role in intensive cultivated soils such as alluvial soils of Indo-Gangetic Plains (IGP). However, it is often identified as deficient micronutrient because of its strong association with soil minerals and low solubility under aerobic and high-pH conditions, Fe is often unavailable to plants, despite its abundance in most soils. Investigating kinetic models along with geochemical model provide a valuable framework for understanding how any nutrient availability shifts over time along with its distribution. In the present investigation, an incubation experiment was carried out with soils of 20 different locations throughout the region at various moisture content to observe the temporal release pattern of Fe. The results indicate that both moisture status and incubation duration significantly influence 0.05 M Ca(NO_3_)_2_-extractable Fe in IGP soils. Soil properties such as pH and EC showed significant negative correlations with amount of Fe release, whereas CEC, clay content, moisture content, and field capacity exhibited significant positive relationships. Moisture plays a key role in regulating redox-driven dissolution and reprecipitation of Fe, while incubation time promotes aging and stabilization of Fe minerals in these soils.

## Introduction

Iron (Fe) is a critical micronutrient for plant physiological processes, including chlorophyll synthesis, respiration, and enzymatic reactions. Despite its abundance in most soils, Fe is frequently unavailable to plants due to its strong association with soil minerals and its low solubility under aerobic and high-pH conditions [1]. The Indo-Gangetic Plains (IGP), considered the food bowl of India due to extensive wheat and rice cultivation, exhibit increasing cases of Fe deficiency in crops, primarily due to intensive agricultural practices, use of high-yielding varieties, and calcareous soil conditions [2,3]. Studies indicate that approximately 12–15% of soils in the region show Fe deficiency symptoms, particularly under paddy cultivation, where redox-induced Fe transformations are critical [4]. The dynamic nature of Fe in soils, governed by factors such as pH, redox potential, organic matter, microbial activity, and the presence of competing ions, makes its behaviour complex and spatially variable. Among the various pools of soil Fe, the soil solution fraction represents the most immediately available form to plants, and its concentration fluctuates rapidly with changing moisture and aeration conditions, especially in flooded and upland rice systems [5,6]. The soil solution Fe primarily present as Fe^2+^ under reducing conditions and Fe^3+^ under oxidizing conditions and is the most immediate source for plant uptake. Its concentration, however, is highly dynamic and influenced by fluctuations in soil moisture, temperature, redox potential (Eh), and organic carbon levels [7,8]. These biogeochemical interactions highlight the need for a kinetic approach to studying Fe transformations, as static measurements of total Fe content do not accurately reflect its plant-available forms [9].

Investigating kinetic models provides a valuable framework for understanding how nutrient availability shifts over time [10]. Knowledge of the kinetics of release under such fluctuating conditions is vital for the maintenance of a sustainable crop yield and the efficient use of fertilizers. The complexity of the Fe cycle in alluvial soils necessitates the use of a change in approach from static extraction methods to the use of a kinetic approach. Static methods such as the measurement of the amount of DTPA-extractable Fe provide a ‘snapshot’ of the situation and do not take into account the rate-limiting steps in the replenishment of the soil solution pool from the solid phase. A multi-model kinetic analysis provides a powerful tool for the measurement and mechanism of the release rates. Due to the complex interactions among soil constituents and the presence of various reactive sorption sites, a range of kinetic approaches such as different order-based models, the Elovich equation, power function, and parabolic diffusion are frequently utilized to characterize the release of nutrients like Fe from the solid phase into the soil solution [10–12]. Applying these models facilitates a detailed evaluation and comparison of nutrient release behaviour under different soil conditions, offering deeper insights into the mechanisms involved. Although these kinetic models are widely used to describe nutrient release processes, no single model can universally explain the release behaviour of native Fe across diverse soils, as the dominant mechanisms like dissolution of Fe-bearing minerals, surface desorption, and diffusion varies with soil physicochemical properties and moisture conditions [10]. Kinetic studies involving time-dependent extraction and modelling provide a more accurate representation of Fe release mechanisms, accounting for both rapid and slow-release fractions [13]. These approaches help decipher the contribution of diffusion-controlled processes, mineral dissolution, and microbial mediation in Fe solubilization, particularly under alternating aerobic-anaerobic field conditions typical of IGP soils. Recent findings suggest that soil moisture plays a pivotal role in Fe release, with higher extractability under moist conditions due to partial reduction of Fe(III) to the more soluble Fe(II) form [14,15].

Another way of evaluation of the chemistry of soil Fe is the use of thermodynamic geochemical models. Although kinetic models provide important information on the rate and mechanism of nutrient release, they do not provide a comprehensive understanding of the chemical forms of nutrients in the soil solution. The chemical speciation of Fe in the soil solution is a function of several factors such as pH, ionic strength, and concentration of carbonate and other complexing ligands [7,8]. Geochemical models such as Visual MINTEQ are commonly used to simulate aqueous chemical speciation reactions and to predict the stability of aqueous metal complexes and solid phases. Using such models, it is possible to estimate the chemical species of Fe and its potential to form solid phases in the soil solution. The use of kinetic models along with geochemical speciation analysis can provide a comprehensive understanding of nutrient behaviour in the soil system. Kinetic models can be used to understand the dynamics of Fe release into the soil solution, while geochemical models such as Visual MINTEQ can be used to understand the chemical speciation of Fe in the soil solution.

Despite its significance, limited research has focused on the kinetic and geochemical models of Fe release into the soil solution in the Indo-Gangetic context. Understanding Fe kinetics is vital for developing more accurate prediction models of nutrient availability and for designing Fe management strategies tailored to region-specific soil and climatic conditions. Moreover, kinetic and thermodynamic data can inform fertilizer recommendations, guide foliar Fe applications, and improve varietal screening for Fe efficiency in staple crops like rice and wheat. Hence, in the present study we aimed to investigate a comprehensive understanding of Fe availability in the soil system and its behaviour with kinetic as well as thermodynamic approach. By integrating laboratory and environmental parameters, this research seeks to advance our understanding of Fe bioavailability and its controlling mechanisms in one of India’s most agriculturally significant regions.

## Materials and methods

### Location of soil samples

A total of twenty bulk surface (0–15 cm) soil samples were collected from cultivated and non-cultivated fields of different locations of IGP covering 5 states and 1 union territory of the country (Table 1). All the collected soil samples are alluvial soils belonging to Inceptisol soil order. The collected soil samples were air dried in shade; grounded in wooden mortar and pestle and sieved through 2 mm sieve and stored in plastic bags and further were used for laboratory experiment.

**Table 1:** Details of the soil sampling locations.

Soil	Location	State/ UT	Latitude	Longitude
S1	IARI-Farm	Delhi	28.64085	77.15474
S2	Samastipur	Bihar	25.98333	85.68333
S3	Hisar	Haryana	29.15915	75.62738
S4	Ludhiana	Punjab	30.5359	75.4741
S5	Gurugram	Haryana	28.39613	76.7777
S6	Patna	Bihar	25.3531	85.0507
S7	Varanasi	Uttar Pradesh	25.32471	82.95631
S8	Karnal	Haryana	29.70596	76.9737
S9	Rewari	Haryana	28.30243	76.69703
S10	Nawada	Bihar	24.88527	85.53024
S11	Kalyani	West Bengal	22.9868	88.45397
S12	Modipuram	Uttar Pradesh	29.06615	77.69168
S13	Patiala	Punjab	30.26822	76.38967
S14	Barrakpore	West Bengal	22.75995	88.40163
S15	Lucknow-2	Uttar Pradesh	26.82705	80.97551
S16	Madhyamgram	West Bengal	22.67655	88.47028
S17	Lucknow-1	Uttar Pradesh	26.90812	80.75071
S18	IARI-Kaveri	Delhi	28.62961	77.16482
S19	Madanpur Khadar-1	Delhi	28.52974	77.32749
S20	Madanpur Khadar-2	Delhi	28.52849	77.32557

### Characterization of experimental soil samples

Soil pH and electrical conductivity (EC) were assessed using a 1:2 soil-to-water suspension. A glass electrode pH meter (Systronics Model 361) and an EC meter (Systronics Model 306) were employed for these measurements [16]. Soil texture was classified using the Bouyoucos hydrometer technique [17], followed by interpretation via the USDA textural triangle. To evaluate field capacity (at 0.033 MPa), a pressure plate extractor (Soil Moisture Equipment Corp., Santa Barbara, CA, USA) [18]. Organic carbon content was determined after passing soil through a 0.2 mm sieve, using the wet oxidation method [19]. Standard protocols were followed to analyze available nitrogen, phosphorus, and potassium [16,20,21]. Cation exchange capacity (CEC) was estimated using the ammonium acetate method [16]. Micronutrient availability, including Fe, was evaluated after extraction with 0.005 M DTPA, 0.01 M CaCl_2_, and 0.1 M triethanolamine (TEA) (pH 7.3) in 1:2 soil: solution ratio and 2 hrs shaking time, and quantified using flame atomic absorption spectroscopy (Spectrum Z-Xpress 8000) [22]. DTPA is a strong chelating agent that effectively complexes micronutrient cations while the TEA buffer maintains a slightly alkaline pH (≈7.3), minimizing dissolution of non-labile mineral phases and providing an estimate of plant-available Fe. Free calcium carbonate (CaCO_3_) was quantified using the rapid titration technique [23]. Rapid titration for free CaCO_3_ was employed because it provides a rapid and reliable estimation of carbonate content through acid neutralization, making it suitable for routine analysis of calcareous and alluvial soils. Additionally, free sesquioxides were extracted using the sodium dithionite–sodium citrate method [16], and the concentrations of Feand aluminum in the extract were analyzed using inductively coupled plasma mass spectrometry (ICP-MS, PerkinElmer NexION 300X). The method is widely accepted for quantifying free sesquioxides in soils where sodium dithionite serves as a powerful reducing agent that dissolves crystalline free Fe oxides, while sodium citrate complexes the released Fe ions and prevents their re-precipitation during extraction.

### Incubation experiment

An incubation experiment was carried out to study the temporal release behaviour of native Fe in the experimental soils at ICAR-Indian Agricultural Research Institute, New Delhi to examine the impact of various moisture contents on Fe release from the soils. For this purpose, 50g air dried soil samples were taken in a polypropylene bottle and various moisture content namely FC (Field capacity), 0.75FC, 0.5FC and 0.25FC were maintained. Following that, the soil samples were placed in an incubator (REMI CIS-24 PLUS) at 25°C for the duration of 2, 4, 7, 15, 30 and 45 days after incubation (DAI). Each sample was replicated thrice. After the desired incubation period, the soil samples were extracted with 0.05 M Ca(NO_3_)_2_ by maintain 1:2 soil:extractant ratio for 2 hours in environmental shaker. The extraction procedure was carried out in closed polypropylene bottles the caps were opened only for addition of the extracting reagent and recapped immediately without prolonged exposure to air. After filtration with Whatman No 42 filter paper, Fe content in the filtrate were measured through AAS (Spectrum Z-Xpress 8000).

### Geochemical model

The geochemical speciation of Fe in soil extracts was simulated using Visual MINTEQ (version 4.0.11), a widely used equilibrium-based chemical speciation model. The model was employed to assess the distribution of Fe among aqueous species, free ions, and potential solid phases. The input parameters are soil solution pH, free CaCO_3_ content, and concentrations of extracting ions and Ca(NO_3_)_2_-extractable Fe content at various moisture contents (0.25FC and FC) and various incubation period (2 DAI and 45 DAI). The system was defined assuming equilibrium conditions, and appropriate databases embedded within Visual MINTEQ were used with Debye-Huckel activity correction was employed to account for complexation reactions, ion activities, and mineral solubility equilibria. Redox conditions were approximated based on moisture status to evaluate Fe^2+^/Fe^3+^ speciation dynamics. The model outputs provided insights into dominant Fe species, saturation indices (SI) of Fe-bearing minerals, and the influence of soil physicochemical properties on Fe mobility. The Saturation Index (SI) is used to evaluate whether the extracted soil solution is undersaturated, saturated, or supersaturated with respect to a mineral phase using the following formula:


$$\begin{array}{c} SI=\text{log}\left(\frac{IAP}{K_{s}}\right) \end{array}$$

IAP: Ion Activity Product

K_s_: Solubility product constant of the mineral

The concentrations of Fe and other dissolved constituents used as inputs to Visual MINTEQ were obtained from experimentally measured total dissolved concentrations determined by FAAS/ICP-MS. These analytical techniques quantify total elemental concentrations in solution and do not directly identify or quantify individual aqueous chemical species where a physical separation step is required prior to detection. Visual MINTEQ utilizes these measured total concentrations together with the measured solution chemistry like pH and ionic composition, to calculate the equilibrium distribution of dissolved Fe species based on thermodynamic principles. Therefore, it is important to note that the predicted Fe species represent model-derived equilibrium speciation estimates rather than experimentally measured chemical species.

### Kinetic models

The application of kinetic models provide insight into the time dependent Fe release kinetics for desorption process. The release of 0.05 M Ca(NO_3_)_2_-extractable Fe with time was fitted into several linear kinetic models, such as the Elovich, power function, parabolic diffusion, pseudo-first order, and pseudo-second order equations. The mathematical equation associated with the models are enlisted as follows:

**Table:** 

Model	Mathematical equation
Zero order	q_t_=q_0_+k_0_t
First order	ln q_t_ = ln q_0_− k_1_t
Second order	1/q_t_ = 1/q_0_ − k_2_t
Pseudo second order	t/q_t_=1/k_2_(q_e_)^2^+t/q_e_
Power function	ln qt = ln b + k_f_ (ln t)
Parabolic diffusion	q_t_ = a + k_p_t^1/2^
Elovich	q_t_ = 1/β ln(αβ) + (1/β) ln t

qt and q_o_ represent the quantities of metal released by the chelating agent at extraction time t (min) and at the initial time point (t = 0), respectively. The parameters k^0^, k_1_ and k_2_ denote the rate constants associated with zero, first and second-order kinetic models (min^−1^), k_p_ is the diffusion rate constant (mg kg^−1^) ^−0.5^, k_f_ is the rate coefficient value (mg kg^−1^ min^−1^) and ‘a’ and ‘b’ are empirical constants. In Elovich equation α represents the initial release rate (mg kg^−1^ min^−1^) and β represents the rate constant (mg kg^−1^ min^−1^).

Zero-order kinetics describes nutrient release at a constant rate over time, independent of the nutrient concentration remaining in the soil, while first-order kinetics assumes that the release rate is directly proportional to the concentration left. The Elovich model, initially proposed by Roginsky and Zeldovich [24], was developed to describe gas adsorption on heterogeneous surfaces by accounting for variations in activation energy, particularly where this energy increases with surface coverage. This model has since been extended to various surface-mediated reactions, including those involving nutrient dynamics in soils. The power function model, as introduced by Dalal [25], was originally used to explain ion release via anion exchange resins and has since been applied to nutrient mobilization from organic and mineral sources, as well as in weathering studies [26]. The parabolic diffusion model explains the kinetics of ion movement in clays and soils, typically where diffusion is the rate-limiting step [27]. The pseudo-first-order model, introduced by Lagergren [28], assumes that physical adsorption governs the process, with the rate dependent on the difference between the adsorption capacity and the amount already adsorbed, making it widely applicable for sorption studies. In contrast, the pseudo-second-order model, proposed by Ho and McKay, is based on the idea that chemisorption is the controlling mechanism and that adsorption is related to the number of available active sites, distinguishing kinetic behaviour in terms of solid-phase adsorption capacity rather than solution concentration [29].

### Statistical analysis

The data were statistically analysed by Least Significant difference, completely randomized design, Pearson correlation, and step-wise regression analyses using statistical procedure [30] with the help of the R software using doebioresearch [31] and agricolae package [32]. The test of significance was conducted at p ≤ 0.001, p ≤ 0.01 and p ≤ 0.05.

## Result

### Initial Physiochemical Properties of experimental soils

**Fig 1:**
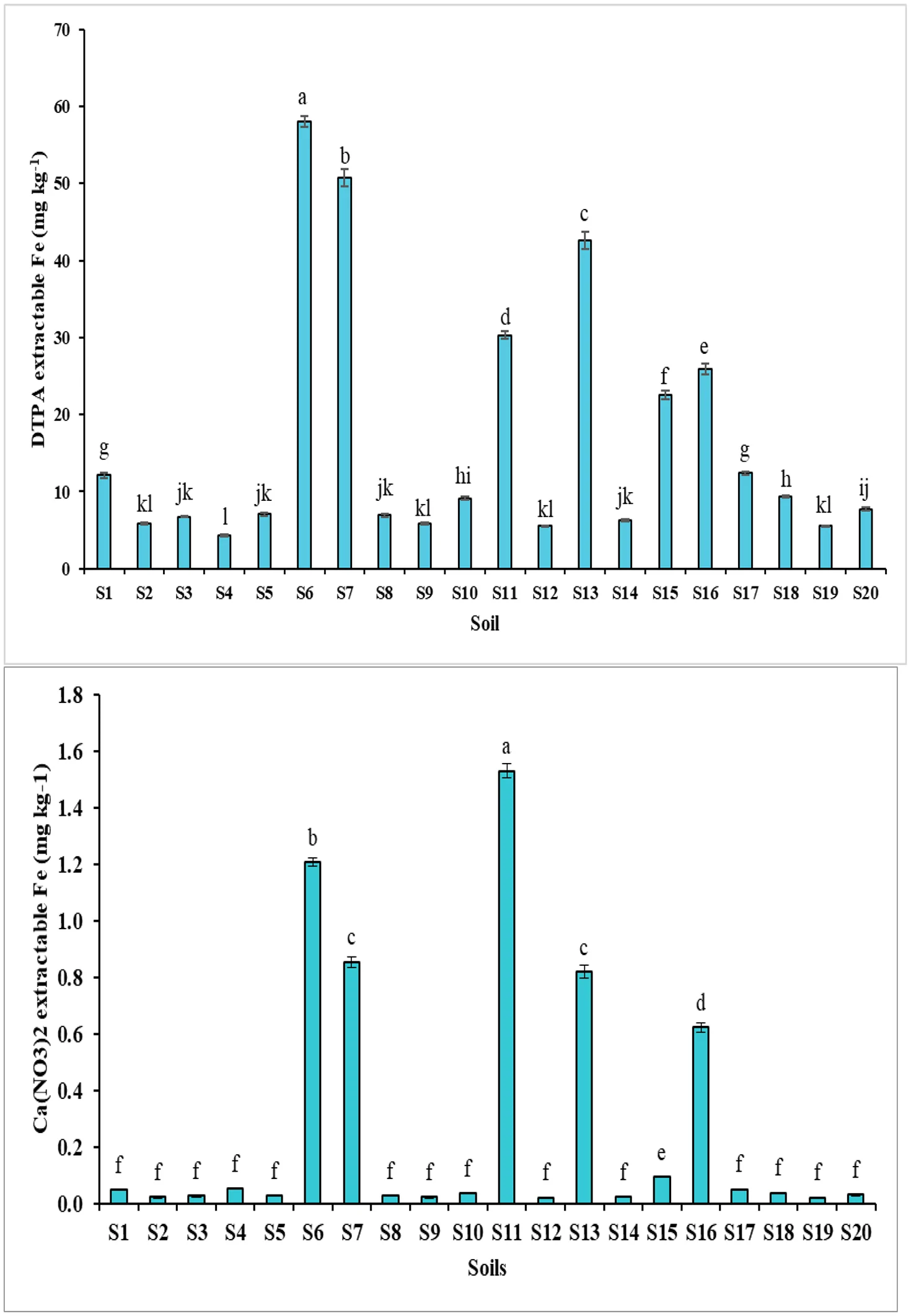
Initial DTPA and 0.05 M Ca(NO_3_)_2_ extractable iron content in experimental soils.

The physicochemical properties of the collected soil samples showed significant variability (Table 2). The texture of the soils varied from clay loam to sandy loam. The sand content varied from 15.5% (S6) to 77.7% (S4), while the content of silt varied from 6% (S19) to 50% (S13), and clay content varied from 10% (S2) to 42.7% (S6). The field capacity varied from a minimum of 18.3% (S4) to a maximum of 36.2% (S11). The range of electrical conductivity varied from 0.09 dS m^−1^ (S4 and S11) to 0.79 dS m^−1^ (S8). The pH of the soils varied from moderately acidic (6.6 in S11) to strongly alkaline (9.1 in S8). The content of CaCO_3_ was the maximum in S2 (33.3%), while the remaining soils did not contain significant amounts of CaCO_3_. The content of organic carbon varied from 0.25% (S15) to 0.77% (S10). The cation exchange capacity varied from 3.8 Cmol kg^−1^ (S4) to 22 Cmol kg^−1^ (S9). Free Fe and Al oxides varied from 0.83 to 2.71 g kg^−1^ and 2.11 to 3.51 g kg^−1^. The DTPA extractable Fe content in the collected soils ranged from 4.31 to 58.07 mg kg^−1^ whereas, the content of 0.05 M Ca(NO_3_)_2_ extractable Fe varied from 0.023 to 1.53 mg kg^−1^ (Fig 1).

**Table 2:** Initial Physical and chemical properties of experimental soils.

Soil	Sand (%)	Silt (%)	Clay (%)	Texture	FC (%)	EC (dS m^-1^)	pH	CaCO_3_ (%)	OC (%)	CEC (Cmol/kg)
S1	53.7	21.7	24.6	SCL	25.5	0.20 ± 0.005 gh	7.9 ± 0.26 bcd	0.71 ± 0.02 ghi	0.55 ± 0.01 c	14 ± 0.37 e
S2	55	35	10.0	SL	21.5	0.24 ± 0.005 f	8.3 ± 0.25 ab	33.3 ± 0.63 a	0.42 ± 0.01 fg	5.2 ± 0.10 i
S3	58.4	18	23.6	SCL	24.5	0.19 ± 0.002 ghi	8.1 ± 0.21 bc	1.64 ± 0.01 de	0.29 ± 0.01 k	13 ± 0.11 f
S4	77.7	10	12.3	SL	18.3	0.09 ± 0.004 k	6.7 ± 0.39 fg	0.38 ± 0.02 hij	0.35 ± 0.02 ij	3.8 ± 0.17 j
S5	71.7	14	14.3	SL	19.8	0.74 ± 0.019 b	8.0 ± 0.26 bc	1.97 ± 0.05 cd	0.37 ± 0.01 hij	13 ± 0.33 f
S6	15.5	41.8	42.7	SIC	26.7	0.33 ± 0.004 e	7.2 ± 0.18 cdefg	0.10 ± 0.01 j	0.47 ± 0.01 de	15 ± 0.18 de
S7	50.4	26	23.6	SCL	25.7	0.20 ± 0.004 gh	7.7 ± 0.26 bcde	0.78 ± 0.02 gh	0.48 ± 0.01 d	12 ± 0.26 fg
S8	53.7	24	22.3	SCL	24.8	0.79 ± 0.029 a	9.1 ± 0.42 a	1.32 ± 0.05 ef	0.48 ± 0.02 d	11 ± 0.42 g
S9	66	16	18.0	SL	21.7	0.17 ± 0.003 hij	7.5 ± 0.27 bcdefg	0.35 ± 0.01 hij	0.30 ± 0.01 k	22 ± 0.44 a
S10	48.4	34	17.6	L	24.2	0.22 ± 0.005 fg	7.7 ± 0.2 bcde	0.50 ± 0.01 hij	0.77 ± 0.02 a	16 ± 0.38 c
S11	21	42	37.0	CL	36.2	0.10 ± 0.002 k	6.6 ± 0.17 g	0.24 ± 0.01 ij	0.34 ± 0.01 j	20 ± 0.33 b
S12	53.7	28	18.3	SL	23.6	0.21 ± 0.003 fg	8.2 ± 0.19 b	0.23 ± 0.01 ij	0.44 ± 0.01 ef	15 ± 0.19 cd
S13	23	50	27.0	CL	31.3	0.31 ± 0.008 e	8.0 ± 0.23 bcd	2.37 ± 0.06 bc	0.30 ± 0.01 k	19 ± 0.50 b
S14	55.7	27.2	17.1	SL	22.9	0.17 ± 0.004 hij	7.1 ± 0.24 defg	0.24 ± 0.01 ij	0.38 ± 0.01 hi	8.9 ± 0.19 h
S15	46	33	21.0	L	25.5	0.16 ± 0.004 ij	7.6 ± 0.23 bcdef	1.04 ± 0.03 fg	0.25 ± 0.01 l	12 ± 0.30 fg
S16	21.4	44.6	31.0	CL	28.6	0.19 ± 0.006 ghi	6.9 ± 0.29 efg	0.16 ± 0.01 j	0.43 ± 0.01 fg	11 ± 0.33 g
S17	32	49	19.0	L	27.4	0.20 ± 0.003 gh	7.7 ± 0.22 bcde	0.47 ± 0.01 hij	0.27 ± 0.01 kl	12 ± 0.20 g
S18	53.7	21.7	24.6	SCL	25.5	0.15 ± 0.002 j	8.2 ± 0.24 b	1.81 ± 0.02 de	0.40 ± 0.01 gh	14 ± 0.18 de
S19	77.3	6	16.6	SL	19.9	0.63 ± 0.006 c	7.9 ± 0.12 bcd	2.54 ± 0.02 b	0.62 ± 0.01 b	9.4 ± 0.09 h
S20	69.8	10.4	19.8	SL	29.5	0.57 ± 0.015 d	7.3 ± 0.28 cdefg	0.43 ± 0.01 hij	0.64 ± 0.02 b	12 ± 0.31 fg
Range				CL-SL	18.3–36.2	0.09–0.74	6.6–9.1	0.10–33.3	0.25–0.77	3.8–22

### Temporal release of 0.05 M Ca(NO_3_)_2_ extractable Fe

**Fig 2:**
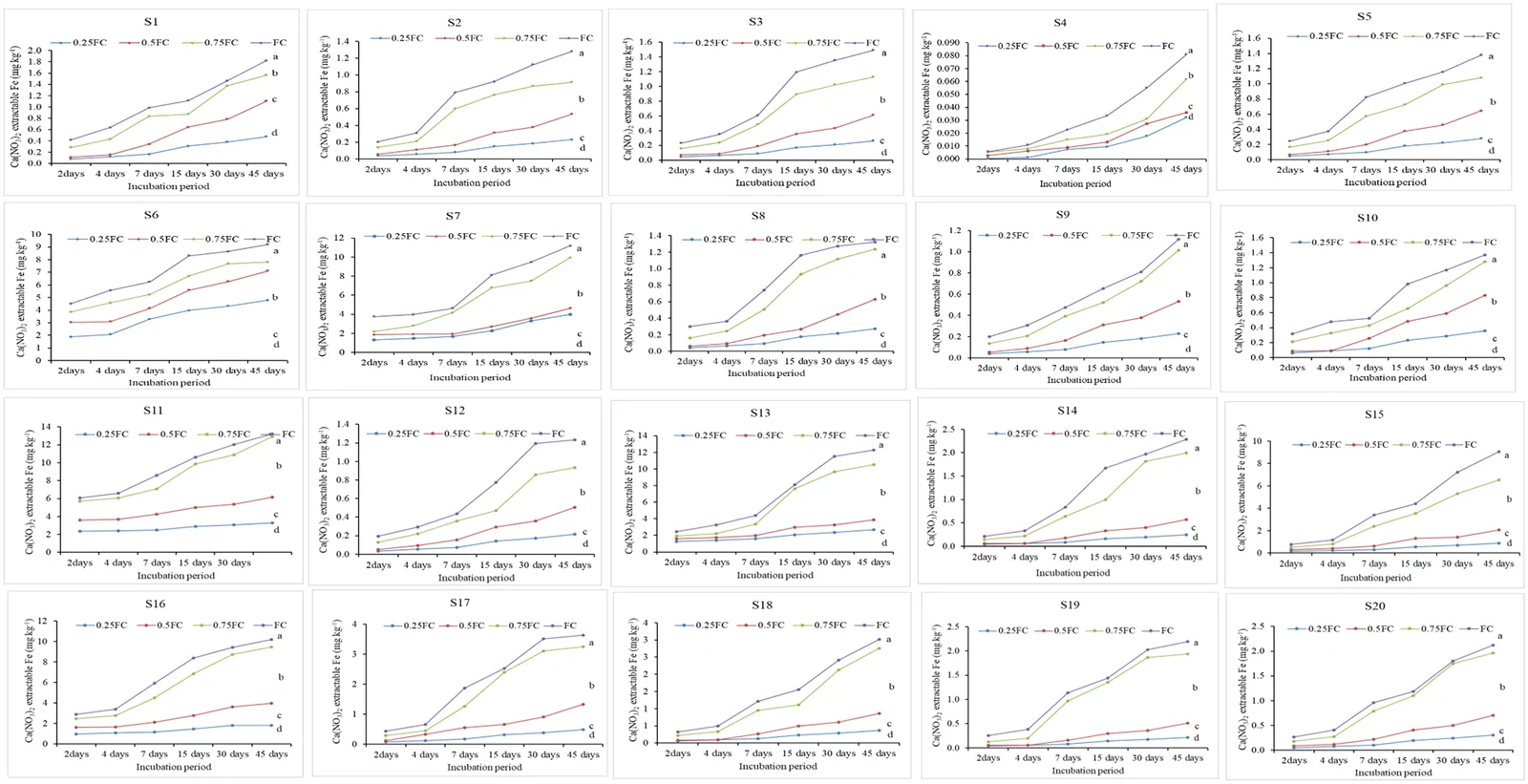
Release pattern of 0.05 M Ca(NO_3_)_2_ extractable iron in experimental soils (S1-S20) as influenced by different moisture contents.

Moisture content has a prominent and significant impact on the amount of 0.05 M Ca(NO_3_)_2_ extractable Fe content in the experimental soils (Fig 2). A significantly higher 0.05 M Ca(NO_3_)_2_ extractable Fe content (2.83 mg kg^-1^) was observed in the soils incubated with FC amount of water content while a minimum of 0.74 mg kg^-1^ was observed in the soils treated with 0.25FC amount of water content. The 0.05 M Ca(NO_3_)_2_ extractable Fe content was also influenced by soil type. The maximum amount of 0.05 M Ca(NO_3_)_2_ extractable Fe content was observed in S11 (1.53 mg kg^-1^) followed by S6 (1.21 mg kg^-1^), S7 (0.86 mg kg^-1^), S13 (0.86 mg kg^-1^) and S16 (0.62 mg kg^-1^). There was a gradual and significant increase in 0.05 M Ca(NO_3_)_2_ extractable Fe after 4 days after incubation (DAI) and a maximum of 2.92 mg kg^-1^ was observed 45 DAI.

### Relationship between amount of iron released and different soil properties

A significant and negative impact of pH and EC was observed on the amount of 0.05 M Ca(NO_3_)_2_ extractable Fe content. However, parameters like CEC, Clay content, moisture content, FC and DTPA extractable Fe content was found to have positive and significant correlation with the quantity of Fe released (Table 3). A stepwise regression analysis of the correlated parameters with the amount of Fe released indicate that a 74% variability in the quantity of Fe released can be explained by pH, moisture content and clay content of the experimental soils. A detail of the relationship between amount of Fe released and different soil properties are represented in Table 4.

**Table 3:** Correlation coefficients between amount of iron released (mg kg^-1^) and different soil properties.

Soil properties	Correlation coefficient (r)
pH	– 0.38**
Electrical conductivity	– 0.27*
Cation exchange capacity	0.48**
Clay	0.71**
Moisture content	0.69**
Field capacity	0.75**
DTPA extractable Fe	0.77**

** Correlation is significant at the 0.01 level (2-tailed); * Correlation is significant at the 0.05 level (2-tailed).

**Table 4:** Stepwise regression equation between amount of iron released (mg kg^-1^) and different soil properties.

Stepwise Regression equation	R^2^	F value
Fe released = 2.00–0.76 pH + 0.13 Moisture content + 0.15 Clay	0.74	54.2***

*** Correlation is significant at the 0.001 level (2-tailed)

### Geochemical speciation of Fe in experimental soils

#### Impact of variable moisture condition on saturation behaviour of Fe.

**Fig 3:**
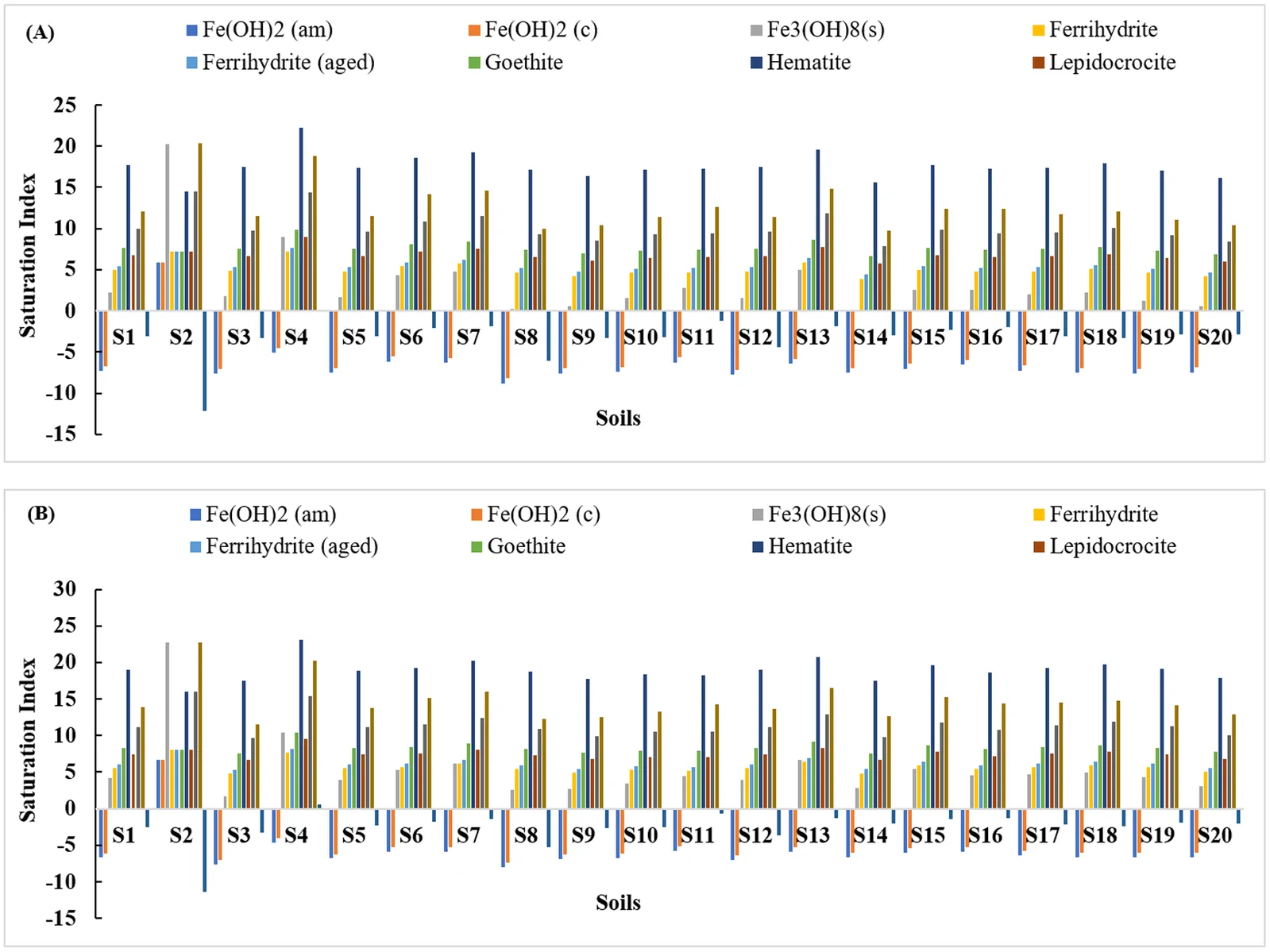
Impact of various moisture content [(A) 0.25FC and (B) FC] on the saturation index values of different iron bearing minerals in experimental soils.

Saturation index values show marked differences in thermodynamic stability of Fe-bearing minerals at different locations and under varying moisture conditions (Fig 3). The Fe^2+^ minerals, i.e., Fe(OH)_2_ (amorphous and crystalline), show negative values of SI, varying from −3.99 to −8.79, indicating undersaturation and dissolution tendencies under both the moisture conditions in all the soils except S2. In contrast, Fe^3+^ minerals such as ferrihydrite, aged ferrihydrite, goethite, hematite, and lepidocrocite show positive values of SI, indicating supersaturation and precipitation tendencies. Among these, hematite and magnetite show the maximum degree of supersaturation, followed by goethite and ferrihydrite, indicating their major role in controlling Fe solubility. The SI values show marked effects of varying moisture conditions on mineral stability. Field capacity (FC) conditions show higher values of SI compared to 0.25 FC, especially for ferrihydrite, goethite, hematite, and magnetite, indicating higher precipitation or lower solubility tendencies at higher moisture conditions. However, Fe(OH)_2_ minerals show undersaturation tendencies under both moisture conditions, though slightly higher under FC conditions. Spatial variability is also marked by differences in SI values at different locations. In S2 show relatively higher values of SI for some of the Fe^3+^ minerals, indicating higher supersaturation tendencies, whereas soils collected from S9, relatively lower values of SI were observed for some of the Fe^3+^ minerals. Siderite is consistently undersaturated except S4, indicating lower possibilities of precipitation of this mineral. The SI values show that iron solubility and precipitation tendencies in Indo-Gangetic alluvial soils are controlled by precipitation-dissolution equilibria of Fe^3+^ oxide minerals and moisture status plays an important role in controlling these reactions.

#### Impact of incubation duration on saturation behaviour of Fe.

**Fig 4:**
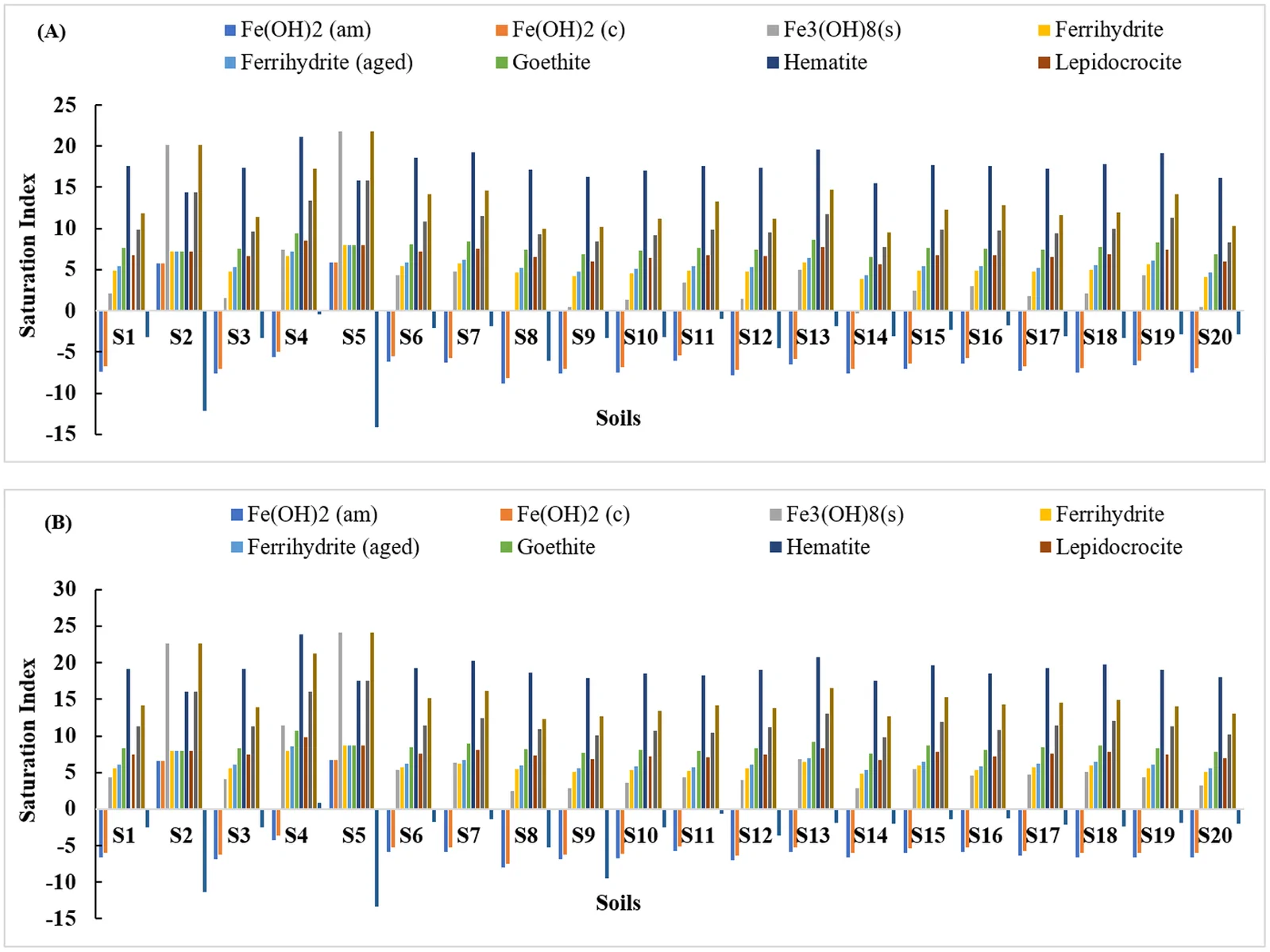
Impact of various incubation duration [(A) 2 days and (B) 45 days] on the saturation index values of different iron bearing minerals in experimental soils.

The SI values clearly reflect the influence of incubation time on the thermodynamic properties of Fe-bearing minerals in different soils (Fig 4). In the case of Fe^2+^ minerals like Fe(OH)_2_ (amorphous and crystalline), have shown consistent negative values of SI at both 2 days and 45 DAI except S2 and S5, suggesting undersaturated conditions w.r.t the Fe^2+^ minerals and a greater potential for dissolution. Although there are slight increases in SI at 45 DAI in most of the soils, these phases are still far from equilibrium, suggesting low stability of reduced Fe species under these conditions. In contrast, ferrihydrite, aged ferrihydrite, goethite, hematite, and lepidocrocite, which are composed of Fe^3+^ ions, showed consistent positive SI values at different stages of incubation, suggesting high levels of supersaturation and a high potential for precipitation. It is interesting to note that SI values of these Fe oxide phases were generally higher at 45 DAI compared to 2 DAI, suggesting increasing stability of these Fe oxide phases in the experimental soil samples over the incubation period. Hematite and magnetite showed the higher levels of supersaturation compared to goethite and ferrihydrite, the maximum SI values of the former Fe^3+^ minerals were 23.4 and 24.2, respectively. These results clearly reflect the dominant role of these Fe oxide phases in governing Fe solubility in different soil samples. Moreover, the effect of incubation time was evident in intermediate Fe phases such as Fe_3_(OH)_8_, in which saturation index values were significantly higher at 45 DAI in most of the soil samples, suggesting increasing levels of transformation towards stable Fe species. In ferrihydrite and aged ferrihydrite, SI values were consistently higher at 45 DAI compared to 2 DAI, suggesting increasing levels of precipitation in these Fe oxide phases. In terms of soil samples, S2 and S5 showed relatively high SI values in different Fe oxide phases at both stages of incubation compared to other soil samples, suggesting high levels of supersaturation in these soil samples. In contrast, S8 and S14 showed low SI values in different Fe oxide phases. In contrast, SI values of siderite were consistently negative in all soil samples at both stages of incubation except S4 after 45 DAI, suggesting low potential for formation under the prevailing conditions. Overall, in experimental soils, increasing incubation duration enhances precipitation and reduces the mobility of Fe, resulting in enhanced precipitation.

### Kinetic analysis of Fe release from different soils

A number of mathematical models were evaluated to determine how effectively they describe Fe release from different soils in the IGP. The comparison of and Akaike Information Criterion (AIC) and root mean square error (RMSE) (Table 5) were considered as primary and secondary criterion for best fitted model selection whereas, coefficient of determination (R^2^) values were evaluated for additional confirmation. The RMSE measures prediction error between observed and predicted values whereas, AIC measures model quality while penalizing complexity to prevent overfitting. The model with the lowest AIC and RMSE along with the highest adjusted R^2^ was considered the best fit. Among the experimental soils, Fe release in eight soils followed parabolic diffusion kinetic model where as Evolich, second order and zero kinetics were followed by six, five and one soils, respectively. The power function kinetic model suited best for the higher moisture status (FC and 0.75FC) while at lower moisture status (0.5FC and 0.25FC) parabolic diffusion was found to be the most fitted model (Table 6).

**Table 5:** Parameters of best fitted linear kinetic models in different soils of IGP.

Soil	Model	R^2^	AIC Value	RMSE	Slope	Intercept
S1	Parabolic Diffusion	0.983	−13.9	0.046	0.186	0.003
S2	Elovich	0.971	−15.8	0.039	0.209	−0.050
S3	Parabolic Diffusion	0.935	−8.44	0.073	0.146	−0.046
S4	Zero order	0.988	−52.300	0.002	0.001	0.003
S5	Elovich	0.983	−17.7	0.033	0.234	−0.061
S6	Second Order	0.698	−17.8	0.033	−0.003	0.259
S7	Second Order	0.790	−12.3	0.053	−0.007	0.387
S8	Elovich	0.975	−14.4	0.044	0.249	−0.080
S9	Parabolic Diffusion	0.984	−20.3	0.027	0.111	−0.039
S10	Parabolic Diffusion	0.987	−18.2	0.032	0.149	−0.040
S11	Second Order	0.798	−24.4	0.019	−0.002	0.208
S12	Parabolic Diffusion	0.989	−21.3	0.025	0.123	−0.066
S13	Second Order	0.717	−6.53	0.085	−0.009	0.456
S14	Elovich	0.974	−8.66	0.071	0.400	−0.278
S15	Parabolic Diffusion	0.989	0.98	0.159	0.800	−0.674
S16	Second Order	0.666	−7.48	0.079	−0.007	0.419
S17	Elovich	0.983	−4.99	0.097	0.674	−0.357
S18	Parabolic Diffusion	0.990	−11.4	0.057	0.299	−0.256
S19	Elovich	0.973	−8.94	0.070	0.381	−0.214
S20	Parabolic Diffusion	0.983	−11.9	0.054	0.217	−0.138

**Table 6:** Parameters of best fitted linear kinetic models in various moisture content across different soils of IGP.

Moisture status	Model	R^2^	AIC Value	RMSE	Slope	Intercept
FC	Power Function	0.977	−7.88	0.076	0.453	−0.124
0.75FC	Power Function	0.976	−6.77	0.083	0.488	−0.402
0.5FC	Parabolic Diffusion	0.982	−10.5	0.061	0.240	0.298
0.25FC	Parabolic Diffusion	0.982	−17.7	0.034	0.131	0.261

## Discussions

### Initial physiochemical properties of experimental soils

The rice-wheat system of the Indo-Gangetic Plain is of significant importance for food security in South Asia. In this region, Fe status is characterized by significant spatial variability over a wide range of agro-climatic zones [33] which is also evidenced by the DTPA as well as Ca(NO_3_)_2_ extractable Fe content of the experimental soils. The changes in physical and chemical characteristics show the heterogeneous nature of the soil in the studied IGP regions. For example, the high FC in clay-rich soil samples like S11 clearly shows the impact of clay content in improving soil FC. Similarly, the increased EC in some samples (S5, S8, S19, and S20) indicates the presence of different amounts of soluble salts in these samples, which may point to localized areas of slightly saline conditions. While most of the studied soil samples are non-calcareous with low to moderate amounts of OC, the unusually high concentration of CaCO_3_ in sample S2 indicates a localized environment with a high concentration of carbonate. The high CEC in some soil samples (e.g., S6, S11, S13, and S16), which are rich in clay content, indicates a high surface area and high charge density in these samples. However, the high CEC in Soil S9, which has a sandy loam texture, indicates the role of specific mineralogical characteristics in improving soil exchange capacity. On the other hand, S2, was found to have exceptionally high levels of calcium carbonate content along with a low cation exchange capacity of 5.2 cmol kg^−1^, thereby indicating that excessive levels of carbonate content do not imply a greater level of nutrient retention. Soils S6 and S11 were found to have both high levels of clay content and cation exchange capacity, thereby indicating a greater potential for nutrient retention. Soils like S4 and S5, which are of lighter texture, were found to have a moderate level of field capacity, which could be due to the presence of silt content. From the above results, it is evident that a wide range of fertility status is associated with the soils of the samples, which could have a significant impact on agricultural productivity. Soils like S10, S13, and S19 are found to have a high level of organic carbon content along with a moderate to high level of cation exchange capacity, thereby indicating a greater potential for agricultural productivity. Soils like S4 and S2, which are of sandy and alkaline nature, may require amendments for better productivity.

### Relationship between amount of iron released and different soil properties

Previous studies have shown that micronutrients, like Fe, as extracted by the DTPA method, are strongly affected by soil pH and organic carbon, which show considerable variability from the arid regions of the west to the humid regions of the east [34]. Higher soil pH and calcium carbonate (CaCO_3_) concentration are two significant factors that affect Fe mobility and Fe release rates. An increase in pH causes Fe precipitation in the form of hydroxides and adsorption on soil particles. According to various studies, the self-diffusion coefficient of Feis significantly reduced in soils with high pH and concentration of CaCO_3_ [35,36]. A seventy-fold decrease in the diffusion coefficient was observed with an increase of pH 4.2 to 7.9 in alluvial soil of Ludhiana [37]. In our experiment, the amount of Fe released was found to have a negative and significant correlation with pH and EC of the soils. The significant positive correlation between clay content with the amount of 0.05 M Ca(NO_3_)_2_ may be linked by the higher moisture retentive capacity and higher diffusion coefficient as soil moisture can affect the rate of diffusion by influencing the cross-sectional area, tortuosity, and the ability of water to move in proximity to the clay surface [37]. For a given moisture content, the self-diffusion coefficient of the ion in the soil increases with an increase in the proportion of ions in the soil solution with regard to the total ions in the soil [38].

### Kinetic analysis of Fe release from different soils

The basic principle of parabolic diffusion model is Fick’s laws of diffusion, formulated by Adolf Fick in 1855 [39]. Its application to solid-liquid interfaces in chemistry was developed in the early-to-mid 20^th^ century. When temporal Fe release data fits in the parabolic diffusion model well, it indicates a transport-controlled mechanism. This means the rate-limiting step is the diffusion of Fe ions through a depletion zone. As Fe is released from the surface of a soil particle, a weathered layer or a layer of secondary precipitates forms and for more Fe to be released, it must diffuse through this thickening layer, which is why the rate of release slows down in proportion to the square root of time. The Elovich model was originally developed by S.Z. Roginsky and Y.B. Zeldovich in 1934 and later popularized by S.Y. Elovich in 1939 to describe the chemisorption of gases onto solid surfaces [40]. In 1980, Chien and Clayton [41] successfully adapted the Elovich equation to describe nutrient (phosphate) release and sorption in soils. It has since become a staple for modeling the release of micronutrients like Fe. Unlike models that assume a uniform soil surface, a good fit to the Elovich equation indicates surface variability. This means the soil has different types of Fe-binding sites, each needing a different amount of energy for release. As the easily accessible Fe (with low activation energy) is released first, the remaining Fe requires more energy to be released and hence, the overall reaction rate decreases exponentially as the reaction continues. The true second-order kinetics have been a fundamental part of physical chemistry since the late 19th century. However, the Pseudo-Second-Order model specifically used for solid-liquid adsorption/desorption was popularized by Y.S. Ho and G. McKay in 1999 [29]. Fitting of the temporal Fe release data in true second-order model in some soils indicate a bimolecular collision mechanism. This means the rate-limiting step requires two molecules in the liquid phase to physically collide and react. It is important to note that the kinetic relationships developed in this study are primarily applicable to the surface agricultural soil layer (0–15 cm), which represents the principal root zone and the soil depth most relevant for crop nutrient uptake, soil fertility assessment, and fertilizer management. Because the objective of the study was to evaluate Fe dynamics under contrasting moisture conditions in soils of agricultural land, deeper soil horizons were not included. However, subsoil layers may differ substantially in physicochemical properties, including redox potential, organic matter content, mineralogical composition, clay content, carbonate content, and moisture retention characteristics, all of which can influence Fe dissolution, precipitation, and release kinetics. Consequently, the kinetic parameters reported here should not be directly extrapolated to deeper soil horizons without appropriate validation. Future investigations incorporating complete soil profiles under varying hydrological conditions would provide a more comprehensive understanding of depth-dependent Fe release kinetics and geochemical speciation, thereby improving the broader applicability of the proposed models.

### Geochemical speciation of Fe in experimental soils

Evaluation of SI values for different moisture condition and incubation periods provides significant insights into the thermodynamic and temporal factors controlling Fe dynamics in IGP alluvial soils. The consistent undersaturation of Fe^2+^-containing phases such as Fe(OH)_2_, under both varying moisture regimes and incubation periods, confirms that these Fe^2+^-containing compounds are thermodynamically unstable and hence more likely to dissolve. This observation aligns with established understanding that Fe^2+^ dominates under reducing environments but is rapidly oxidized under oxic or fluctuating conditions, limiting its stability in most agricultural soils [41]. In contrast, consistently positive SI values for Fe^3+^-containing phases such as ferrihydrite, goethite, hematite, and lepidocrocite indicate that these Fe^3+^-containing compounds are supersaturated and hence have tendency of precipitation. This is consistent with the well-accepted notion that Fe solubility is largely regulated by Fe (III) (oxyhydr) oxides, which act as primary sinks for dissolved Fe [42]. Ferrihydrite is known to be a short-range-ordered Fe^3+^ compound, whereas goethite and hematite are more ordered and stable. Previous studies have shown that ferrihydrite is known to dominate fine soil textures, whereas goethite and hematite become more significant as these Fe-containing compounds become more stable and aged [43]. This is consistent with the higher SI values obtained for goethite and hematite, as obtained in the current study.

Moisture content of the soil played important role affecting Fe saturation behaviour. The general increase of SI under FC compared to 0.25 FC indicates an increase in order of Fe^3+^ minerals. Soil moisture is an important factor affecting the oxidation-reduction potential of the soil. Ferrihydrite exposed to higher water activity nucleates and grows hematite domains more readily, indicating water is a key driver of crystallization from the ferrihydrite precursor [44]. Water promotes transformation primarily by enabling dissolution–reprecipitation and by supplying the mobility needed for nucleation and growth of crystalline domains [45]. Incubation duration is another factor affecting Fe transformation as SI values of Fe^3+^ minerals are generally higher at 45 DAI than at 2 DAI. The increase of SI values of Fe^3+^ minerals indicate the transformation of short-range ordered Fe^3+^ phases into stable Fe^3+^ phases. Such mineralogical transformations are well documented, where the transformation of ferrihydrite into goethite or hematite occurs through dissolution and reprecipitation mechanisms [46]. In this study, the increasing values of SI for aged ferrihydrite, goethite, and hematite has further confirmed the aging and crystallization mechanisms during incubation. Furthermore, the increasing values of SI for intermediate phases such as Fe_3_(OH)_8_ suggest the transitional mechanisms in Fe oxide formation. The undersaturation of siderite in all conditions indicates a low possibility for Fe carbonate formation, possibly due to insufficient reducing conditions and/or insufficient carbonate. The spatial variability in Fe oxide phases among the investigated sites further confirmed the impact of intrinsic soil properties such as pH, texture, organic carbon content, and mineralogy on Fe oxide formation. Soils such as S2 and S5 showed higher values of SI, indicating a higher potential for Fe oxide formation. The geochemical models such as Visual MINTEQ as used for the present study provide valuable mechanistic insights into the equilibrium distribution and potential behaviour of dissolved Fe species in soil solutions. However, validation of the predicted Fe species was beyond the scope of the present investigation. Instruments like FAAS or ICP-MS without prior physical separation techniques, measures total dissolved Fe concentrations rather than individual chemical species. Therefore, the modelled Fe speciation of this study represents thermodynamic predictions based on measured solution chemistry and should be interpreted as mechanistic estimates rather than experimentally verified species. Future studies with dedicated analytical speciation techniques like HPLC-ICP-MS is required for direct validation of the simulated species.

## Conclusion

The obtained results suggest that there was a significant impact of moisture status and incubation period on 0.05 M Ca(NO_3_)_2_ extractable iron content in the soils of IGP. Soil properties like pH and EC has significant and negative impact whereas, CEC, Clay content, moisture content, FC has positive and significant correlation with the quantity of iron released. Iron oxide phases in these alluvial soils are controlled by a combination of thermodynamic equilibrium and kinetic transformation mechanisms. Moisture status plays a crucial role in redox-driven dissolution and reprecipitation mechanisms, whereas incubation time is responsible for aging and stabilization. Furthermore, the dominant Fe^3+^ oxide phases indicate their potential role in controlling iron and nutrient interaction and the long-term geochemistry of these soils.

## Supporting information

S1 File Raw data on Initial DTPA and 0.05 M Ca(NO_3_)_2_ extractable iron content and effect of moisture status and incubation duration on saturation index of different iron bearing minerals. (DOCX)
