# Supplementary material for: Iron release dynamics in Indo-Gangetic alluvial soils under variable moisture conditions: Insights from multi-model kinetic and geochemical speciation analysis
Source: PLoS One. 2026 Aug 28;21(8):e0355846. doi: 10.1371/journal.pone.0355846 (PMC13524240; doi:10.1371/journal.pone.0355846)
Supplement: Supplementary file 1 [file pone.0355846.s002.pdf]

The temporal release kinetics and geochemical speciation of native Iron (Fe) in Indo-Gangetic Alluvial Soils under varying moisture conditions. The combination of mechanistic kinetic equations with thermodynamic equilibrium modeling (Visual MINTEQ) provides a robust platform to evaluate micronutrient dynamics. However, the paper requires critical modifications to resolve inconsistencies in the text, clarify data parameters, and strengthen the overall scientific validity.

Line-by-Line Specific Comments

1. **Lines 27–29:** The text states, "...an incubation experiment was carried out with soils of **20 different locations**..." , but Line 108 in the Materials and Methods section explicitly states, "A total of **twenty-five bulk surface** (0-15 cm) soil samples were collected...". This inconsistency must be resolved. Additionally, Table 1 only lists 20 soils (S1 to S20). Please correct the text to reflect the exact number of soils evaluated.

2. **Line 74:** Clarify what "specific compounds" refers to in this context. Because this paper focuses exclusively on native iron (Fe) release, frame this sentence specifically around iron minerals or desorption behavior to make it contextually relevant.

3. **Lines 80–81:** The text notes that higher extractability occurs due to the partial reduction of Fe(III) to Fe(II). Because Ca(NO_3)_2 is an unbuffered salt extractant used to evaluate the water-soluble/exchangeable pool, please state whether any precautions (such as purging with inert gas or immediate acidification) were taken during filtration to prevent the rapid oxidation and precipitation of Fe{2+} back into Fe{3+} oxyhydroxides.

4. **Lines 109–110:** The phrase "...covering 5 states and 1 union territory..." should be explicitly verified against the contents of Table 1. Table 1 lists locations from Delhi, Bihar, Haryana, Punjab, and West Bengal, which comprises 4 states and 1 Union Territory (Delhi). Please correct the tally of states.

5. **Lines 126–128:** It is mentioned that micronutrient availability was evaluated using "0.005 M DTPA, 0.01 M CaCl_2, and 0.1 M triethanolamine (TEA)". This standard Lindsay and Norvell extractant mixture uses **0.005 M DTPA, 0.05 M $CaCl_2, and 0.1 M TEA**. Please double-check if the concentration of CaCl_2 was indeed 0.01 M or if this is a typographical error.

6. **Line 138:** The text indicates that moisture contents of "FC, 0.75FC, 0.5FC and 0.25FC were implied". The correct scientific terminology here should be **"applied"** or **"maintained"** rather than "implied".

7. **Lines 141–142:** Explain the rationale behind selecting **0.05 M Ca(NO_3)_2** as the extracting agent for time-dependent kinetics. While it effectively targets exchangeable fractions without shifting the matrix excessively, comparison with traditional DTPA or water-soluble pools should be justified to demonstrate its physiological relevance to plant uptake.

8. **Line 153:** The text mentions that the **"Debye-Huckel activity correction"** was used. Given that soil solution extracts can vary in ionic strength, specify whether the *Extended Debye-Hückel* or the *Davies equation* was automatically prioritized by Visual MINTEQ based on the calculated ionic strength of the extract matrix.

9. **Line 168 (Kinetic Models Table):** * In the First-order equation and Second-order equation , the minus sign indicates a *desorption/decay* processes where solution concentration decreases over time. However, the data shows that extractable Fe **increases** over incubation time (from 4 DAI to 45 DAI). Linear forms of accumulation/release equations should feature a positive sign for the rate constants if q_t represents cumulative iron released into the solution phase over time. Please rectify the signs to align with your release data.
    a. For the Elovich equation clearly define the physical meaning of the constants (initial release rate) and (desorption constant) relative to your soil parameters.

10. **Line 190:** The phrase "statistical procedure [30]" is missing its specific package name or programmatic reference within the text. Please explicitly state the R package used (e.g., `agricolae`, `lm`, etc.).

11. **Lines 204–205:** There is a mismatch between the text and the table range summary. Line 204 states that DTPA extractable Fe ranged from **4.31 to 58.07 mg kg^{-1}** , but the reported range row at the bottom of Table 2 (Line 109) omits the DTPA range entirely, showing only **3.8 – 22** for CEC. Ensure all parameters discussed in the text are fully summarized within the table boundaries.

Recommended Literature for Enhancing Quality

Reviewing the following publications will help improve the interpretation and the validation of redox-driven mineral speciation transformations:

https://doi.org/10.1016/j.cej.2009.01.014 ,

https://doi.org/10.1016/j.geoderma.2022.115842, https://doi.org/10.1002/ldr.5446

https://doi.org/10.1002/eco.2662
