# Supplementary material for: Iron release dynamics in Indo-Gangetic alluvial soils under variable moisture conditions: Insights from multi-model kinetic and geochemical speciation analysis
Source: PLoS One. 2026 Aug 28;21(8):e0355846. doi: 10.1371/journal.pone.0355846 (PMC13524240; doi:10.1371/journal.pone.0355846)
Supplement: Supplementary file 2 [file pone.0355846.s001.docx]

Table S1: Raw values of dataset represented in Figure 1

| Initial DTPA-extractable Fe content (mg kg^-1^): | | | |
| --- | --- | --- | --- |
| R1 | R2 | R3 | Mean |
| 11.49 | 11.98 | 12.82 | 12.10 |
| 5.63 | 5.86 | 6.10 | 5.86 |
| 6.57 | 6.77 | 6.77 | 6.70 |
| 3.88 | 4.36 | 4.70 | 4.31 |
| 6.70 | 7.48 | 6.99 | 7.06 |
| 58.65 | 56.33 | 59.23 | 58.07 |
| 48.18 | 51.22 | 52.75 | 50.72 |
| 7.25 | 6.29 | 7.18 | 6.91 |
| 5.94 | 5.53 | 6.00 | 5.82 |
| 9.09 | 8.64 | 9.55 | 9.09 |
| 30.92 | 30.92 | 29.10 | 30.31 |
| 5.71 | 5.43 | 5.49 | 5.54 |
| 45.09 | 42.12 | 40.41 | 42.54 |
| 6.17 | 5.99 | 6.55 | 6.24 |
| 23.88 | 21.63 | 22.08 | 22.53 |
| 26.43 | 27.20 | 24.10 | 25.91 |
| 12.77 | 12.52 | 11.90 | 12.40 |
| 9.18 | 9.28 | 9.65 | 9.37 |
| 5.45 | 5.56 | 5.67 | 5.56 |
| 8.15 | 7.61 | 7.31 | 7.69 |

| Initial 0.05M Ca(NO_3_)_2_ extractable Fe content (mg kg^-1^): | | | |
| --- | --- | --- | --- |
| R1 | R2 | R3 | Mean |
| 0.048 | 0.050 | 0.053 | 0.05 |
| 0.023 | 0.024 | 0.025 | 0.02 |
| 0.028 | 0.028 | 0.028 | 0.03 |
| 0.048 | 0.054 | 0.058 | 0.05 |
| 0.028 | 0.031 | 0.029 | 0.03 |
| 1.219 | 1.171 | 1.231 | 1.21 |
| 0.812 | 0.863 | 0.889 | 0.85 |
| 0.030 | 0.026 | 0.030 | 0.03 |
| 0.025 | 0.023 | 0.025 | 0.02 |
| 0.038 | 0.036 | 0.040 | 0.04 |
| 1.560 | 1.560 | 1.469 | 1.53 |
| 0.024 | 0.023 | 0.023 | 0.02 |
| 0.870 | 0.812 | 0.779 | 0.82 |
| 0.026 | 0.025 | 0.027 | 0.03 |
| 0.100 | 0.090 | 0.092 | 0.09 |
| 0.635 | 0.654 | 0.579 | 0.62 |
| 0.053 | 0.052 | 0.050 | 0.05 |
| 0.038 | 0.039 | 0.040 | 0.04 |
| 0.023 | 0.023 | 0.024 | 0.02 |
| 0.034 | 0.032 | 0.030 | 0.03 |

Table S2 Effect of moisture status on saturation index of different iron bearing minerals in experimental soils

|  |  | Moisture Status | | | |
| --- | --- | --- | --- | --- | --- |
|  |  | 0.25FC | | FC | |
| Soil | Mineral | SI | log IAP | SI | log IAP |
| S1 | Fe(OH)_2_ (am) | -7.31 | 6.18 | -6.684 | 6.806 |
|  | Fe(OH)_2_ (c) | -6.71 | 6.18 | -6.084 | 6.806 |
|  | Fe_3_(OH)_8_(s) | 2.248 | 22.47 | 4.127 | 24.349 |
|  | Ferrihydrite | 4.945 | 8.145 | 5.572 | 8.772 |
|  | Ferrihydrite (aged) | 5.455 | 8.145 | 6.082 | 8.772 |
|  | Goethite | 7.657 | 8.148 | 8.283 | 8.774 |
|  | Hematite | 17.715 | 16.297 | 18.968 | 17.55 |
|  | Lepidocrocite | 6.777 | 8.148 | 7.403 | 8.774 |
|  | Maghemite | 9.911 | 16.297 | 11.164 | 17.55 |
|  | Magnetite | 12.062 | 22.479 | 13.941 | 24.357 |
|  | Siderite | -3.129 | -14.17 | -2.503 | -13.54 |
| S2 | Fe(OH)_2_ (am) | 5.858 | 5.858 | 6.655 | 6.655 |
|  | Fe(OH)_2_ (c) | 5.858 | 5.858 | 6.655 | 6.655 |
|  | Fe_3_(OH)_8_(s) | 20.297 | 20.297 | 22.689 | 22.689 |
|  | Ferrihydrite | 7.22 | 7.22 | 8.017 | 8.017 |
|  | Ferrihydrite (aged) | 7.22 | 7.22 | 8.017 | 8.017 |
|  | Goethite | 7.226 | 7.226 | 8.023 | 8.023 |
|  | Hematite | 14.458 | 14.458 | 16.052 | 16.052 |
|  | Lepidocrocite | 7.226 | 7.226 | 8.023 | 8.023 |
|  | Maghemite | 14.458 | 14.458 | 16.052 | 16.052 |
|  | Magnetite | 20.321 | 20.321 | 22.713 | 22.713 |
|  | Siderite | -12.124 | -12.124 | -11.327 | -11.327 |
| S3 | Fe(OH)_2_ (am) | -7.613 | 5.877 | -7.613 | 6.669 |
|  | Fe(OH)_2_ (c) | -7.013 | 5.877 | -7.013 | 6.669 |
|  | Fe_3_(OH)_8_(s) | 1.74 | 21.962 | 1.74 | 24.338 |
|  | Ferrihydrite | 4.843 | 8.043 | 4.843 | 8.834 |
|  | Ferrihydrite (aged) | 5.353 | 8.043 | 5.353 | 8.834 |
|  | Goethite | 7.554 | 8.045 | 7.554 | 8.837 |
|  | Hematite | 17.509 | 16.091 | 17.509 | 17.675 |
|  | Lepidocrocite | 6.674 | 8.045 | 6.674 | 8.837 |
|  | Maghemite | 9.705 | 16.091 | 9.705 | 17.675 |
|  | Magnetite | 11.553 | 21.97 | 11.553 | 24.346 |
|  | Siderite | -3.293 | -14.332 | -3.293 | -13.54 |
| S4 | Fe(OH)_2_ (am) | -5.074 | 8.416 | -4.594 | 8.896 |
|  | Fe(OH)_2_ (c) | -4.474 | 8.416 | -3.994 | 8.896 |
|  | Fe_3_(OH)_8_(s) | 8.959 | 29.181 | 10.396 | 30.618 |
|  | Ferrihydrite | 7.182 | 10.382 | 7.661 | 10.861 |
|  | Ferrihydrite (aged) | 7.692 | 10.382 | 8.171 | 10.861 |
|  | Goethite | 9.893 | 10.384 | 10.373 | 10.864 |
|  | Hematite | 22.189 | 20.771 | 23.148 | 21.73 |
|  | Lepidocrocite | 9.013 | 10.384 | 9.493 | 10.864 |
|  | Maghemite | 14.385 | 20.771 | 15.344 | 21.73 |
|  | Magnetite | 18.773 | 29.19 | 20.211 | 30.628 |
|  | Siderite | 0.102 | -10.937 | 0.58 | -10.459 |
| S5 | Fe(OH)_2_ (am) | -7.564 | 5.926 | -6.815 | 6.675 |
|  | Fe(OH)_2_ (c) | -6.964 | 5.926 | -6.215 | 6.675 |
|  | Fe_3_(OH)_8_(s) | 1.689 | 21.911 | 3.934 | 24.156 |
|  | Ferrihydrite | 4.792 | 7.992 | 5.541 | 8.741 |
|  | Ferrihydrite (aged) | 5.302 | 7.992 | 6.051 | 8.741 |
|  | Goethite | 7.503 | 7.994 | 8.252 | 8.743 |
|  | Hematite | 17.409 | 15.991 | 18.906 | 17.488 |
|  | Lepidocrocite | 6.623 | 7.994 | 7.372 | 8.743 |
|  | Maghemite | 9.605 | 15.991 | 11.102 | 17.488 |
|  | Magnetite | 11.502 | 21.919 | 13.748 | 24.165 |
|  | Siderite | -3.042 | -14.081 | -2.293 | -13.333 |
| S6 | Fe(OH)_2_ (am) | -6.149 | 7.341 | -5.828 | 7.662 |
|  | Fe(OH)_2_ (c) | -5.549 | 7.341 | -5.228 | 7.662 |
|  | Fe_3_(OH)_8_(s) | 4.334 | 24.556 | 5.295 | 25.517 |
|  | Ferrihydrite | 5.407 | 8.607 | 5.728 | 8.928 |
|  | Ferrihydrite (aged) | 5.917 | 8.607 | 6.238 | 8.928 |
|  | Goethite | 8.118 | 8.609 | 8.439 | 8.93 |
|  | Hematite | 18.639 | 17.221 | 19.279 | 17.861 |
|  | Lepidocrocite | 7.238 | 8.609 | 7.559 | 8.93 |
|  | Maghemite | 10.835 | 17.221 | 11.475 | 17.861 |
|  | Magnetite | 14.147 | 24.564 | 15.108 | 25.525 |
|  | Siderite | -2.065 | -13.104 | -1.744 | -12.784 |
| S7 | Fe(OH)_2_ (am) | -6.323 | 7.167 | -5.857 | 7.633 |
|  | Fe(OH)_2_ (c) | -5.723 | 7.167 | -5.257 | 7.633 |
|  | Fe_3_(OH)_8_(s) | 4.812 | 25.034 | 6.208 | 26.43 |
|  | Ferrihydrite | 5.733 | 8.933 | 6.199 | 9.399 |
|  | Ferrihydrite (aged) | 6.243 | 8.933 | 6.709 | 9.399 |
|  | Goethite | 8.444 | 8.935 | 8.91 | 9.401 |
|  | Hematite | 19.291 | 17.873 | 20.222 | 18.804 |
|  | Lepidocrocite | 7.564 | 8.935 | 8.03 | 9.401 |
|  | Maghemite | 11.487 | 17.873 | 12.418 | 18.804 |
|  | Magnetite | 14.626 | 25.043 | 16.022 | 26.438 |
|  | Siderite | -1.874 | -12.914 | -1.409 | -12.448 |
| S8 | Fe(OH)_2_ (am) | -8.794 | 4.696 | -8.021 | 5.469 |
|  | Fe(OH)_2_ (c) | -8.194 | 4.696 | -7.421 | 5.469 |
|  | Fe_3_(OH)_8_(s) | 0.198 | 20.42 | 2.516 | 22.738 |
|  | Ferrihydrite | 4.662 | 7.862 | 5.435 | 8.635 |
|  | Ferrihydrite (aged) | 5.172 | 7.862 | 5.945 | 8.635 |
|  | Goethite | 7.373 | 7.864 | 8.146 | 8.637 |
|  | Hematite | 17.148 | 15.73 | 18.694 | 17.276 |
|  | Lepidocrocite | 6.493 | 7.864 | 7.266 | 8.637 |
|  | Maghemite | 9.344 | 15.73 | 10.89 | 17.276 |
|  | Magnetite | 10.012 | 20.428 | 12.33 | 22.747 |
|  | Siderite | -6.018 | -17.057 | -5.245 | -16.285 |
| S9 | Fe(OH)_2_ (am) | -7.595 | 5.895 | -6.908 | 6.582 |
|  | Fe(OH)_2_ (c) | -6.995 | 5.895 | -6.308 | 6.582 |
|  | Fe_3_(OH)_8_(s) | 0.593 | 20.815 | 2.655 | 22.877 |
|  | Ferrihydrite | 4.26 | 7.46 | 4.948 | 8.148 |
|  | Ferrihydrite (aged) | 4.77 | 7.46 | 5.458 | 8.148 |
|  | Goethite | 6.972 | 7.463 | 7.659 | 8.15 |
|  | Hematite | 16.345 | 14.927 | 17.719 | 16.301 |
|  | Lepidocrocite | 6.092 | 7.463 | 6.779 | 8.15 |
|  | Maghemite | 8.541 | 14.927 | 9.915 | 16.301 |
|  | Magnetite | 10.407 | 20.824 | 12.468 | 22.885 |
|  | Siderite | -3.282 | -14.321 | -2.594 | -13.634 |
| S10 | Fe(OH)_2_ (am) | -7.412 | 6.078 | -6.787 | 6.703 |
|  | Fe(OH)_2_ (c) | -6.812 | 6.078 | -6.187 | 6.703 |
|  | Fe_3_(OH)_8_(s) | 1.543 | 21.765 | 3.42 | 23.642 |
|  | Ferrihydrite | 4.643 | 7.843 | 5.269 | 8.469 |
|  | Ferrihydrite (aged) | 5.153 | 7.843 | 5.779 | 8.469 |
|  | Goethite | 7.355 | 7.846 | 7.98 | 8.471 |
|  | Hematite | 17.111 | 15.693 | 18.363 | 16.945 |
|  | Lepidocrocite | 6.475 | 7.846 | 7.1 | 8.471 |
|  | Maghemite | 9.307 | 15.693 | 10.559 | 16.945 |
|  | Magnetite | 11.356 | 21.773 | 13.233 | 23.65 |
|  | Siderite | -3.16 | -14.199 | -2.534 | -13.573 |
| S11 | Fe(OH)_2_ (am) | -6.262 | 7.228 | -5.723 | 7.767 |
|  | Fe(OH)_2_ (c) | -5.662 | 7.228 | -5.123 | 7.767 |
|  | Fe_3_(OH)_8_(s) | 2.794 | 23.016 | 4.411 | 24.633 |
|  | Ferrihydrite | 4.694 | 7.894 | 5.233 | 8.433 |
|  | Ferrihydrite (aged) | 5.204 | 7.894 | 5.743 | 8.433 |
|  | Goethite | 7.405 | 7.896 | 7.944 | 8.435 |
|  | Hematite | 17.212 | 15.794 | 18.29 | 16.872 |
|  | Lepidocrocite | 6.525 | 7.896 | 7.064 | 8.435 |
|  | Maghemite | 9.408 | 15.794 | 10.486 | 16.872 |
|  | Magnetite | 12.608 | 23.024 | 14.225 | 24.641 |
|  | Siderite | -1.185 | -12.225 | -0.646 | -11.686 |
| S12 | Fe(OH)_2_ (am) | -7.736 | 5.754 | -6.964 | 6.526 |
|  | Fe(OH)_2_ (c) | -7.136 | 5.754 | -6.364 | 6.526 |
|  | Fe_3_(OH)_8_(s) | 1.572 | 21.794 | 3.886 | 24.108 |
|  | Ferrihydrite | 4.82 | 8.02 | 5.591 | 8.791 |
|  | Ferrihydrite (aged) | 5.33 | 8.02 | 6.101 | 8.791 |
|  | Goethite | 7.531 | 8.022 | 8.303 | 8.794 |
|  | Hematite | 17.464 | 16.046 | 19.007 | 17.589 |
|  | Lepidocrocite | 6.651 | 8.022 | 7.423 | 8.794 |
|  | Maghemite | 9.66 | 16.046 | 11.203 | 17.589 |
|  | Magnetite | 11.386 | 21.803 | 13.7 | 24.117 |
|  | Siderite | -4.413 | -15.452 | -3.642 | -14.681 |
| S13 | Fe(OH)_2_ (am) | -6.457 | 7.033 | -5.889 | 7.601 |
|  | Fe(OH)_2_ (c) | -5.857 | 7.033 | -5.289 | 7.601 |
|  | Fe_3_(OH)_8_(s) | 5.009 | 25.231 | 6.712 | 26.934 |
|  | Ferrihydrite | 5.899 | 9.099 | 6.467 | 9.667 |
|  | Ferrihydrite (aged) | 6.409 | 9.099 | 6.977 | 9.667 |
|  | Goethite | 8.61 | 9.101 | 9.178 | 9.669 |
|  | Hematite | 19.622 | 18.204 | 20.758 | 19.34 |
|  | Lepidocrocite | 7.73 | 9.101 | 8.298 | 9.669 |
|  | Maghemite | 11.818 | 18.204 | 12.954 | 19.34 |
|  | Magnetite | 14.822 | 25.239 | 16.526 | 26.942 |
|  | Siderite | -1.851 | -12.89 | -1.283 | -12.322 |
| S14 | Fe(OH)_2_ (am) | -7.562 | 5.928 | -6.593 | 6.897 |
|  | Fe(OH)_2_ (c) | -6.962 | 5.928 | -5.993 | 6.897 |
|  | Fe_3_(OH)_8_(s) | -0.105 | 20.117 | 2.802 | 23.024 |
|  | Ferrihydrite | 3.894 | 7.094 | 4.863 | 8.063 |
|  | Ferrihydrite (aged) | 4.404 | 7.094 | 5.373 | 8.063 |
|  | Goethite | 6.605 | 7.096 | 7.574 | 8.065 |
|  | Hematite | 15.613 | 14.195 | 17.551 | 16.133 |
|  | Lepidocrocite | 5.725 | 7.096 | 6.694 | 8.065 |
|  | Maghemite | 7.809 | 14.195 | 9.747 | 16.133 |
|  | Magnetite | 9.709 | 20.126 | 12.615 | 23.032 |
|  | Siderite | -2.993 | -14.033 | -2.025 | -13.064 |
| S15 | Fe(OH)_2_ (am) | -7.012 | 6.478 | -6.05 | 7.44 |
|  | Fe(OH)_2_ (c) | -6.412 | 6.478 | -5.45 | 7.44 |
|  | Fe_3_(OH)_8_(s) | 2.543 | 22.765 | 5.43 | 25.652 |
|  | Ferrihydrite | 4.944 | 8.144 | 5.906 | 9.106 |
|  | Ferrihydrite (aged) | 5.454 | 8.144 | 6.416 | 9.106 |
|  | Goethite | 7.655 | 8.146 | 8.617 | 9.108 |
|  | Hematite | 17.711 | 16.293 | 19.637 | 18.219 |
|  | Lepidocrocite | 6.775 | 8.146 | 7.737 | 9.108 |
|  | Maghemite | 9.907 | 16.293 | 11.833 | 18.219 |
|  | Magnetite | 12.356 | 22.773 | 15.244 | 25.661 |
|  | Siderite | -2.328 | -13.368 | -1.366 | -12.405 |
| S16 | Fe(OH)_2_ (am) | -6.543 | 6.947 | -5.856 | 7.634 |
|  | Fe(OH)_2_ (c) | -5.943 | 6.947 | -5.256 | 7.634 |
|  | Fe_3_(OH)_8_(s) | 2.552 | 22.774 | 4.612 | 24.834 |
|  | Ferrihydrite | 4.713 | 7.913 | 5.4 | 8.6 |
|  | Ferrihydrite (aged) | 5.223 | 7.913 | 5.91 | 8.6 |
|  | Goethite | 7.424 | 7.915 | 8.111 | 8.602 |
|  | Hematite | 17.251 | 15.833 | 18.624 | 17.206 |
|  | Lepidocrocite | 6.544 | 7.915 | 7.231 | 8.602 |
|  | Maghemite | 9.447 | 15.833 | 10.82 | 17.206 |
|  | Magnetite | 12.366 | 22.782 | 14.425 | 24.842 |
|  | Siderite | -1.946 | -12.986 | -1.26 | -12.299 |
| S17 | Fe(OH)_2_ (am) | -7.278 | 6.212 | -6.37 | 7.12 |
|  | Fe(OH)_2_ (c) | -6.678 | 6.212 | -5.77 | 7.12 |
|  | Fe_3_(OH)_8_(s) | 1.946 | 22.168 | 4.669 | 24.891 |
|  | Ferrihydrite | 4.778 | 7.978 | 5.686 | 8.886 |
|  | Ferrihydrite (aged) | 5.288 | 7.978 | 6.196 | 8.886 |
|  | Goethite | 7.489 | 7.98 | 8.397 | 8.888 |
|  | Hematite | 17.38 | 15.962 | 19.196 | 17.778 |
|  | Lepidocrocite | 6.609 | 7.98 | 7.517 | 8.888 |
|  | Maghemite | 9.576 | 15.962 | 11.392 | 17.778 |
|  | Magnetite | 11.76 | 22.176 | 14.483 | 24.9 |
|  | Siderite | -3.052 | -14.092 | -2.144 | -13.184 |
| S18 | Fe(OH)_2_ (am) | -7.507 | 5.983 | -6.624 | 6.866 |
|  | Fe(OH)_2_ (c) | -6.907 | 5.983 | -6.024 | 6.866 |
|  | Fe_3_(OH)_8_(s) | 2.258 | 22.48 | 4.908 | 25.13 |
|  | Ferrihydrite | 5.048 | 8.248 | 5.932 | 9.132 |
|  | Ferrihydrite (aged) | 5.558 | 8.248 | 6.442 | 9.132 |
|  | Goethite | 7.76 | 8.251 | 8.643 | 9.134 |
|  | Hematite | 17.921 | 16.503 | 19.688 | 18.27 |
|  | Lepidocrocite | 6.88 | 8.251 | 7.763 | 9.134 |
|  | Maghemite | 10.117 | 16.503 | 11.884 | 18.27 |
|  | Magnetite | 12.071 | 22.488 | 14.721 | 25.138 |
|  | Siderite | -3.263 | -14.303 | -2.38 | -13.419 |
| S19 | Fe(OH)_2_ (am) | -7.648 | 5.84 | -6.622 | 6.868 |
|  | Fe(OH)_2_ (c) | -7.048 | 5.84 | -6.022 | 6.868 |
|  | Fe_3_(OH)_8_(s) | 1.237 | 21.46 | 4.314 | 24.536 |
|  | Ferrihydrite | 4.608 | 7.81 | 5.634 | 8.834 |
|  | Ferrihydrite (aged) | 5.118 | 7.81 | 6.144 | 8.834 |
|  | Goethite | 7.319 | 7.81 | 8.345 | 8.836 |
|  | Hematite | 17.041 | 15.62 | 19.093 | 17.675 |
|  | Lepidocrocite | 6.439 | 7.81 | 7.465 | 8.836 |
|  | Maghemite | 9.237 | 15.62 | 11.289 | 17.675 |
|  | Magnetite | 11.05 | 21.47 | 14.128 | 24.545 |
|  | Siderite | -2.896 | -13.94 | -1.87 | -12.909 |
| S20 | Fe(OH)_2_ (am) | -7.471 | 6.019 | -6.629 | 6.861 |
|  | Fe(OH)_2_ (c) | -6.871 | 6.019 | -6.029 | 6.861 |
|  | Fe_3_(OH)_8_(s) | 0.566 | 20.788 | 3.093 | 23.315 |
|  | Ferrihydrite | 4.185 | 7.385 | 5.027 | 8.227 |
|  | Ferrihydrite (aged) | 4.695 | 7.385 | 5.537 | 8.227 |
|  | Goethite | 6.896 | 7.387 | 7.738 | 8.229 |
|  | Hematite | 16.194 | 14.776 | 17.879 | 16.461 |
|  | Lepidocrocite | 6.016 | 7.387 | 6.858 | 8.229 |
|  | Maghemite | 8.39 | 14.776 | 10.075 | 16.461 |
|  | Magnetite | 10.38 | 20.797 | 12.907 | 23.324 |
|  | Siderite | -2.856 | -13.895 | -2.014 | -13.053 |

Table S3 Effect of incubation duration on saturation index of different iron bearing minerals in experimental soils

|  |  | Incubation duration | | | |
| --- | --- | --- | --- | --- | --- |
|  |  | 2 DAI | | 45 DAI | |
| Soil | Mineral | SI | log IAP | SI | log IAP |
| S1 | Fe(OH)_2_ (am) | -7.366 | 6.124 | -6.621 | 6.869 |
|  | Fe(OH)_2_ (c) | -6.766 | 6.124 | -6.021 | 6.869 |
|  | Fe_3_(OH)_8_(s) | 2.081 | 22.303 | 4.316 | 24.538 |
|  | Ferrihydrite | 4.889 | 8.089 | 5.635 | 8.835 |
|  | Ferrihydrite (aged) | 5.399 | 8.089 | 6.145 | 8.835 |
|  | Goethite | 7.601 | 8.092 | 8.346 | 8.837 |
|  | Hematite | 17.603 | 16.185 | 19.094 | 17.676 |
|  | Lepidocrocite | 6.721 | 8.092 | 7.466 | 8.837 |
|  | Maghemite | 9.799 | 16.185 | 11.29 | 17.676 |
|  | Magnetite | 11.894 | 22.311 | 14.13 | 24.547 |
|  | Siderite | -3.185 | -14.224 | -2.44 | -13.479 |
| S2 | Fe(OH)_2_ (am) | 5.802 | 5.802 | 6.637 | 6.637 |
|  | Fe(OH)_2_ (c) | 5.802 | 5.802 | 6.637 | 6.637 |
|  | Fe_3_(OH)_8_(s) | 20.129 | 20.129 | 22.635 | 22.635 |
|  | Ferrihydrite | 7.164 | 7.164 | 7.999 | 7.999 |
|  | Ferrihydrite (aged) | 7.164 | 7.164 | 7.999 | 7.999 |
|  | Goethite | 7.17 | 7.17 | 8.005 | 8.005 |
|  | Hematite | 14.346 | 14.346 | 16.016 | 16.016 |
|  | Lepidocrocite | 7.17 | 7.17 | 8.005 | 8.005 |
|  | Maghemite | 14.346 | 14.346 | 16.016 | 16.016 |
|  | Magnetite | 20.153 | 20.153 | 22.659 | 22.659 |
|  | Siderite | -12.18 | -12.18 | -11.345 | -11.345 |
| S3 | Fe(OH)_2_ (am) | -7.662 | 5.828 | -6.821 | 6.669 |
|  | Fe(OH)_2_ (c) | -7.062 | 5.828 | -6.221 | 6.669 |
|  | Fe_3_(OH)_8_(s) | 1.595 | 21.817 | 4.117 | 24.339 |
|  | Ferrihydrite | 4.794 | 7.994 | 5.635 | 8.835 |
|  | Ferrihydrite (aged) | 5.304 | 7.994 | 6.145 | 8.835 |
|  | Goethite | 7.505 | 7.996 | 8.346 | 8.837 |
|  | Hematite | 17.413 | 15.995 | 19.094 | 17.676 |
|  | Lepidocrocite | 6.625 | 7.996 | 7.466 | 8.837 |
|  | Maghemite | 9.609 | 15.995 | 11.29 | 17.676 |
|  | Magnetite | 11.409 | 21.825 | 13.931 | 24.348 |
|  | Siderite | -3.341 | -14.38 | -2.5 | -13.54 |
| S4 | Fe(OH)_2_ (am) | -5.585 | 7.905 | -4.25 | 9.24 |
|  | Fe(OH)_2_ (c) | -4.985 | 7.905 | -3.65 | 9.24 |
|  | Fe_3_(OH)_8_(s) | 7.424 | 27.646 | 11.428 | 31.65 |
|  | Ferrihydrite | 6.671 | 9.871 | 8.005 | 11.205 |
|  | Ferrihydrite (aged) | 7.181 | 9.871 | 8.515 | 11.205 |
|  | Goethite | 9.382 | 9.873 | 10.717 | 11.208 |
|  | Hematite | 21.166 | 19.748 | 23.837 | 22.419 |
|  | Lepidocrocite | 8.502 | 9.873 | 9.837 | 11.208 |
|  | Maghemite | 13.362 | 19.748 | 16.033 | 22.419 |
|  | Magnetite | 17.238 | 27.655 | 21.245 | 31.662 |
|  | Siderite | -0.409 | -11.448 | 0.922 | -10.118 |
| S5 | Fe(OH)_2_ (am) | 5.87 | 5.87 | 6.682 | 6.682 |
|  | Fe(OH)_2_ (c) | 5.87 | 5.87 | 6.682 | 6.682 |
|  | Fe_3_(OH)_8_(s) | 21.742 | 21.742 | 24.179 | 24.179 |
|  | Ferrihydrite | 7.936 | 7.936 | 8.748 | 8.748 |
|  | Ferrihydrite (aged) | 7.936 | 7.936 | 8.748 | 8.748 |
|  | Goethite | 7.938 | 7.938 | 8.75 | 8.75 |
|  | Hematite | 15.878 | 15.878 | 17.503 | 17.503 |
|  | Lepidocrocite | 7.938 | 7.938 | 8.75 | 8.75 |
|  | Maghemite | 15.878 | 15.878 | 17.503 | 17.503 |
|  | Magnetite | 21.75 | 21.75 | 24.187 | 24.187 |
|  | Siderite | -14.137 | -14.137 | -13.325 | -13.325 |
| S6 | Fe(OH)_2_ (am) | -6.157 | 7.333 | -5.819 | 7.671 |
|  | Fe(OH)_2_ (c) | -5.557 | 7.333 | -5.219 | 7.671 |
|  | Fe_3_(OH)_8_(s) | 4.309 | 24.531 | 5.321 | 25.543 |
|  | Ferrihydrite | 5.399 | 8.599 | 5.736 | 8.936 |
|  | Ferrihydrite (aged) | 5.909 | 8.599 | 6.246 | 8.936 |
|  | Goethite | 8.11 | 8.601 | 8.448 | 8.939 |
|  | Hematite | 18.622 | 17.204 | 19.297 | 17.879 |
|  | Lepidocrocite | 7.23 | 8.601 | 7.568 | 8.939 |
|  | Maghemite | 10.818 | 17.204 | 11.493 | 17.879 |
|  | Magnetite | 14.122 | 24.539 | 15.135 | 25.552 |
|  | Siderite | -2.073 | -13.113 | -1.736 | -12.775 |
| S7 | Fe(OH)_2_ (am) | -6.334 | 7.167 | -5.822 | 7.633 |
|  | Fe(OH)_2_ (c) | -5.734 | 7.167 | -5.222 | 7.633 |
|  | Fe_3_(OH)_8_(s) | 4.777 | 25.034 | 6.314 | 26.43 |
|  | Ferrihydrite | 5.722 | 8.933 | 6.234 | 9.399 |
|  | Ferrihydrite (aged) | 6.232 | 8.933 | 6.744 | 9.399 |
|  | Goethite | 8.433 | 8.935 | 8.945 | 9.401 |
|  | Hematite | 19.268 | 17.873 | 20.292 | 18.804 |
|  | Lepidocrocite | 7.553 | 8.935 | 8.065 | 9.401 |
|  | Maghemite | 11.464 | 17.873 | 12.488 | 18.804 |
|  | Magnetite | 14.591 | 25.043 | 16.127 | 26.438 |
|  | Siderite | -1.886 | -12.914 | -1.374 | -12.448 |
| S8 | Fe(OH)_2_ (am) | -8.802 | 4.688 | -8.019 | 5.471 |
|  | Fe(OH)_2_ (c) | -8.202 | 4.688 | -7.419 | 5.471 |
|  | Fe_3_(OH)_8_(s) | 0.175 | 20.397 | 2.524 | 22.746 |
|  | Ferrihydrite | 4.654 | 7.854 | 5.437 | 8.637 |
|  | Ferrihydrite (aged) | 5.164 | 7.854 | 5.947 | 8.637 |
|  | Goethite | 7.365 | 7.856 | 8.148 | 8.639 |
|  | Hematite | 17.133 | 15.715 | 18.699 | 17.281 |
|  | Lepidocrocite | 6.485 | 7.856 | 7.268 | 8.639 |
|  | Maghemite | 9.329 | 15.715 | 10.895 | 17.281 |
|  | Magnetite | 9.988 | 20.405 | 12.337 | 22.754 |
|  | Siderite | -6.026 | -17.065 | -5.243 | -16.282 |
| S9 | Fe(OH)_2_ (am) | -7.651 | 5.839 | -6.823 | 6.667 |
|  | Fe(OH)_2_ (c) | -7.051 | 5.839 | -6.223 | 6.667 |
|  | Fe_3_(OH)_8_(s) | 0.425 | 20.647 | 2.909 | 23.131 |
|  | Ferrihydrite | 4.204 | 7.404 | 5.032 | 8.232 |
|  | Ferrihydrite (aged) | 4.714 | 7.404 | 5.542 | 8.232 |
|  | Goethite | 6.916 | 7.407 | 7.744 | 8.235 |
|  | Hematite | 16.233 | 14.815 | 17.889 | 16.471 |
|  | Lepidocrocite | 6.036 | 7.407 | 6.864 | 8.235 |
|  | Maghemite | 8.429 | 14.815 | 10.085 | 16.471 |
|  | Magnetite | 10.239 | 20.656 | 12.723 | 23.14 |
|  | Siderite | -3.337 | -14.377 | -9.467 | 13.237 |
| S10 | Fe(OH)_2_ (am) | -7.468 | 6.022 | -6.711 | 6.779 |
|  | Fe(OH)_2_ (c) | -6.868 | 6.022 | -6.111 | 6.779 |
|  | Fe_3_(OH)_8_(s) | 1.374 | 21.596 | 3.646 | 23.868 |
|  | Ferrihydrite | 4.587 | 7.787 | 5.345 | 8.545 |
|  | Ferrihydrite (aged) | 5.097 | 7.787 | 5.855 | 8.545 |
|  | Goethite | 7.299 | 7.79 | 8.056 | 8.547 |
|  | Hematite | 16.999 | 15.581 | 18.513 | 17.095 |
|  | Lepidocrocite | 6.419 | 7.79 | 7.176 | 8.547 |
|  | Maghemite | 9.195 | 15.581 | 10.709 | 17.095 |
|  | Magnetite | 11.188 | 21.605 | 13.459 | 23.876 |
|  | Siderite | -3.216 | -14.255 | -2.459 | -13.498 |
| S11 | Fe(OH)_2_ (am) | -6.055 | 7.435 | -5.752 | 7.738 |
|  | Fe(OH)_2_ (c) | -5.455 | 7.435 | -5.152 | 7.738 |
|  | Fe_3_(OH)_8_(s) | 3.416 | 23.638 | 4.325 | 24.547 |
|  | Ferrihydrite | 4.901 | 8.101 | 5.204 | 8.404 |
|  | Ferrihydrite (aged) | 5.411 | 8.101 | 5.714 | 8.404 |
|  | Goethite | 7.612 | 8.103 | 7.915 | 8.406 |
|  | Hematite | 17.627 | 16.209 | 18.233 | 16.815 |
|  | Lepidocrocite | 6.732 | 8.103 | 7.035 | 8.406 |
|  | Maghemite | 9.823 | 16.209 | 10.429 | 16.815 |
|  | Magnetite | 13.23 | 23.646 | 14.138 | 24.555 |
|  | Siderite | -0.978 | -12.018 | -0.675 | -11.715 |
| S12 | Fe(OH)_2_ (am) | -7.792 | 5.698 | -6.942 | 6.548 |
|  | Fe(OH)_2_ (c) | -7.192 | 5.698 | -6.342 | 6.548 |
|  | Fe_3_(OH)_8_(s) | 1.405 | 21.627 | 3.953 | 24.175 |
|  | Ferrihydrite | 4.764 | 7.964 | 5.614 | 8.814 |
|  | Ferrihydrite (aged) | 5.274 | 7.964 | 6.124 | 8.814 |
|  | Goethite | 7.475 | 7.966 | 8.325 | 8.816 |
|  | Hematite | 17.353 | 15.935 | 19.051 | 17.633 |
|  | Lepidocrocite | 6.595 | 7.966 | 7.445 | 8.816 |
|  | Maghemite | 9.549 | 15.935 | 11.247 | 17.633 |
|  | Magnetite | 11.219 | 21.636 | 13.766 | 24.183 |
|  | Siderite | -4.469 | -15.508 | -3.62 | -14.659 |
| S13 | Fe(OH)_2_ (am) | -6.475 | 7.015 | -5.868 | 7.622 |
|  | Fe(OH)_2_ (c) | -5.875 | 7.015 | -5.268 | 7.622 |
|  | Fe_3_(OH)_8_(s) | 4.954 | 25.176 | 6.775 | 26.997 |
|  | Ferrihydrite | 5.881 | 9.081 | 6.487 | 9.687 |
|  | Ferrihydrite (aged) | 6.391 | 9.081 | 6.997 | 9.687 |
|  | Goethite | 8.592 | 9.083 | 9.199 | 9.69 |
|  | Hematite | 19.585 | 18.167 | 20.799 | 19.381 |
|  | Lepidocrocite | 7.712 | 9.083 | 8.319 | 9.69 |
|  | Maghemite | 11.781 | 18.167 | 12.995 | 19.381 |
|  | Magnetite | 14.767 | 25.184 | 16.588 | 27.005 |
|  | Siderite | -1.869 | -12.908 | -1.869 | -12.908 |
| S14 | Fe(OH)_2_ (am) | -7.617 | 5.873 | -6.574 | 6.916 |
|  | Fe(OH)_2_ (c) | -7.017 | 5.873 | -5.974 | 6.916 |
|  | Fe_3_(OH)_8_(s) | -0.272 | 19.95 | 2.859 | 23.081 |
|  | Ferrihydrite | 3.839 | 7.039 | 4.882 | 8.082 |
|  | Ferrihydrite (aged) | 4.349 | 7.039 | 5.392 | 8.082 |
|  | Goethite | 6.55 | 7.041 | 7.593 | 8.084 |
|  | Hematite | 15.501 | 14.083 | 17.589 | 16.171 |
|  | Lepidocrocite | 5.67 | 7.041 | 6.713 | 8.084 |
|  | Maghemite | 7.697 | 14.083 | 9.785 | 16.171 |
|  | Magnetite | 9.541 | 19.958 | 12.672 | 23.089 |
|  | Siderite | -3.049 | -14.089 | -2.006 | -13.045 |
| S15 | Fe(OH)_2_ (am) | -7.043 | 6.447 | -6.021 | 7.469 |
|  | Fe(OH)_2_ (c) | -6.443 | 6.447 | -5.421 | 7.469 |
|  | Fe_3_(OH)_8_(s) | 2.45 | 22.672 | 5.517 | 25.739 |
|  | Ferrihydrite | 4.913 | 8.113 | 5.935 | 9.135 |
|  | Ferrihydrite (aged) | 5.423 | 8.113 | 6.445 | 9.135 |
|  | Goethite | 7.624 | 8.115 | 8.646 | 9.137 |
|  | Hematite | 17.65 | 16.232 | 19.694 | 18.276 |
|  | Lepidocrocite | 6.744 | 8.115 | 7.766 | 9.137 |
|  | Maghemite | 9.846 | 16.232 | 11.89 | 18.276 |
|  | Magnetite | 12.264 | 22.681 | 15.331 | 25.747 |
|  | Siderite | -2.359 | -13.399 | -1.337 | -12.376 |
| S16 | Fe(OH)_2_ (am) | -6.384 | 7.106 | -5.878 | 7.612 |
|  | Fe(OH)_2_ (c) | -5.784 | 7.106 | -5.278 | 7.612 |
|  | Fe_3_(OH)_8_(s) | 3.027 | 23.249 | 4.545 | 24.767 |
|  | Ferrihydrite | 4.872 | 8.072 | 5.378 | 8.578 |
|  | Ferrihydrite (aged) | 5.382 | 8.072 | 5.888 | 8.578 |
|  | Goethite | 7.583 | 8.074 | 8.089 | 8.58 |
|  | Hematite | 17.567 | 16.149 | 18.579 | 17.161 |
|  | Lepidocrocite | 6.703 | 8.074 | 7.209 | 8.58 |
|  | Maghemite | 9.763 | 16.149 | 10.775 | 17.161 |
|  | Magnetite | 12.841 | 23.257 | 14.358 | 24.775 |
|  | Siderite | -1.788 | -12.827 | -1.282 | -12.321 |
| S17 | Fe(OH)_2_ (am) | -7.334 | 6.156 | -6.355 | 7.135 |
|  | Fe(OH)_2_ (c) | -6.734 | 6.156 | -5.755 | 7.135 |
|  | Fe_3_(OH)_8_(s) | 1.778 | 22 | 4.714 | 24.936 |
|  | Ferrihydrite | 4.722 | 7.922 | 5.701 | 8.901 |
|  | Ferrihydrite (aged) | 5.232 | 7.922 | 6.211 | 8.901 |
|  | Goethite | 7.433 | 7.924 | 8.412 | 8.903 |
|  | Hematite | 17.268 | 15.85 | 19.226 | 17.808 |
|  | Lepidocrocite | 6.553 | 7.924 | 7.532 | 8.903 |
|  | Maghemite | 9.464 | 15.85 | 11.422 | 17.808 |
|  | Magnetite | 11.592 | 22.008 | 14.528 | 24.944 |
|  | Siderite | -3.108 | -14.148 | -2.13 | -13.169 |
| S18 | Fe(OH)_2_ (am) | -7.564 | 5.926 | -6.558 | 6.932 |
|  | Fe(OH)_2_ (c) | -6.964 | 5.926 | -5.958 | 6.932 |
|  | Fe_3_(OH)_8_(s) | 2.089 | 22.311 | 5.106 | 25.328 |
|  | Ferrihydrite | 4.992 | 8.192 | 5.998 | 9.198 |
|  | Ferrihydrite (aged) | 5.502 | 8.192 | 6.508 | 9.198 |
|  | Goethite | 7.703 | 8.194 | 8.709 | 9.2 |
|  | Hematite | 17.809 | 16.391 | 19.82 | 18.402 |
|  | Lepidocrocite | 6.823 | 8.194 | 7.829 | 9.2 |
|  | Maghemite | 10.005 | 16.391 | 12.016 | 18.402 |
|  | Magnetite | 11.903 | 22.319 | 14.919 | 25.336 |
|  | Siderite | -3.32 | -14.359 | -2.314 | -13.353 |
| S19 | Fe(OH)_2_ (am) | -6.622 | 6.868 | -6.63 | 6.86 |
|  | Fe(OH)_2_ (c) | -6.022 | 6.868 | -6.03 | 6.86 |
|  | Fe_3_(OH)_8_(s) | 4.314 | 24.536 | 4.289 | 24.511 |
|  | Ferrihydrite | 5.634 | 8.834 | 5.626 | 8.826 |
|  | Ferrihydrite (aged) | 6.144 | 8.834 | 6.136 | 8.826 |
|  | Goethite | 8.345 | 8.836 | 8.337 | 8.828 |
|  | Hematite | 19.093 | 17.675 | 19.076 | 17.658 |
|  | Lepidocrocite | 7.465 | 8.836 | 7.457 | 8.828 |
|  | Maghemite | 11.289 | 17.675 | 11.272 | 17.658 |
|  | Magnetite | 14.128 | 24.545 | 14.103 | 24.519 |
|  | Siderite | -2.892 | -13.932 | -1.878 | -12.918 |
| S20 | Fe(OH)_2_ (am) | -7.512 | 6.019 | -6.574 | 6.861 |
|  | Fe(OH)_2_ (c) | -6.912 | 6.019 | -5.974 | 6.861 |
|  | Fe_3_(OH)_8_(s) | 0.444 | 20.788 | 3.257 | 23.315 |
|  | Ferrihydrite | 4.144 | 7.385 | 5.081 | 8.227 |
|  | Ferrihydrite (aged) | 4.654 | 7.385 | 5.591 | 8.227 |
|  | Goethite | 6.855 | 7.387 | 7.793 | 8.229 |
|  | Hematite | 16.112 | 14.776 | 17.987 | 16.461 |
|  | Lepidocrocite | 5.975 | 7.387 | 6.913 | 8.229 |
|  | Maghemite | 8.308 | 14.776 | 10.183 | 16.461 |
|  | Magnetite | 10.257 | 20.797 | 13.07 | 23.324 |
|  | Siderite | -2.897 | -13.895 | -1.959 | -13.053 |
