# Supplementary material for: Iron release dynamics in Indo-Gangetic alluvial soils under variable moisture conditions: Insights from multi-model kinetic and geochemical speciation analysis
Source: PLoS One. 2026 Aug 28;21(8):e0355846. doi: 10.1371/journal.pone.0355846 (PMC13524240; doi:10.1371/journal.pone.0355846)
Supplement: Supplementary file 3 [file pone.0355846.s003.docx]

To

Editor-in-Chief

PLOS One

Sub: Revised manuscript entitled “Iron Release Dynamics in Indo-Gangetic Alluvial Soils under Variable Moisture Conditions: Insights from Multi-Model Kinetic and Geochemical Speciation Analysis” [PONE-D-26-16816]

Sir,

Thank you very much for your kind comments/suggestions for improving our manuscript. All the comments/suggestions are incorporated in the revised manuscript. The point-wise response to comments/suggestions is given below:

Authors response to **Line-by-Line Specific Comments**

**Reviewer 1:**

1. **Lines 27–29:** The text states, "...an incubation experiment was carried out with soils of 20 different locations...", but Line 108 in the Materials and Methods section explicitly states, "A total of twenty-five bulk surface (0-15 cm) soil samples were collected...".

This inconsistency must be resolved. Additionally, Table 1 only lists 20 soils (S1 to S20). Please correct the text to reflect the exact number of soils evaluated.

**Authors Response**: We thank the reviewer for identifying this inconsistency. A total of 20 number of soil samples were collected from 20 different locations of Indo-Gangetic Plain as mentioned in Table 1 and all the 20 soil samples were evaluated. The inconsistency has been rectified in line number 112 in revised marked-up manuscript.

2. **Line 74:** Clarify what "specific compounds" refers to in this context. Because this paper focuses exclusively on native iron (Fe) release, frame this sentence specifically around iron minerals or desorption behavior to make it contextually relevant.

**Authors Response**: The sentence has been corrected accordingly to make it contextually relevant (line no 72 to 76 in revised marked-up manuscript).

3. **Lines 80–81:** The text notes that higher extractability occurs due to the partial reduction of Fe(III) to Fe(II). Because Ca(NO_3_)_2_ is an unbuffered salt extractant used to evaluate the water-soluble/exchangeable pool, please state whether any precautions (such as purging with inert gas or immediate acidification) were taken during filtration to prevent the rapid oxidation and precipitation of Fe^2+^ back into Fe^3+^ oxyhydroxides.

**Authors Response:** Thank you for this important observation. The extraction procedure was carried out in closed polypropylene bottles the caps were opened only for addition of the extracting reagent and recapped immediately without prolonged exposure to air and then shaking was done using in environmental shaker. No inert gas purging was employed because the objective of the study was to quantify Ca(NO₃)₂-extractable Fe rather than to determine Fe²⁺ and Fe³⁺ individually. This clarification has now been incorporated into the Materials and Methods section (Line No 155-158 in revised marked-up manuscript).

4. **Lines 109–110:** The phrase "...covering 5 states and 1 union territory..." should be explicitly verified against the contents of Table 1. Table 1 lists locations from Delhi, Bihar, Haryana, Punjab, and West Bengal, which comprises 4 states and 1 Union Territory (Delhi). Please correct the tally of states.

**Authors Response:** The 20 soil samples were collected from 5 states and 1 union territory is correct. The 5 states are Bihar (S2, S6 and S10), Haryana (S3, S5, S8 and S9), Punjab (S4 and S13), Uttar Pradesh (S7, S12, S15 and S17) and West Bengal (S11, S14 and S16) and 1 union territory is Delhi (S1, S18, S19 and S20).

5. **Lines 126–128:** It is mentioned that micronutrient availability was evaluated using

"0.005 M DTPA, 0.01 M CaCl_2, and 0.1 M triethanolamine (TEA)". This standard

Lindsay and Norvell extractant mixture uses 0.005 M DTPA, 0.05 M CaCl_2_, and 0.1 M TEA. Please double-check if the concentration of CaCl_2_ was indeed 0.01 M or if this is a typographical error.

**Authors Response:** We have used the composition of the DTPA extractant as per the research article published by Lindsay and Norvell (1978) where the concentration of CaCl_2_ is 0.01 M.

Reference: Lindsay, W. L., & Norvell, W. (1978). Development of a DTPA soil test for zinc, iron, manganese, and copper. *Soil science society of America journal*, *42*(3), 421-428. <https://doi.org/10.2136/sssaj1978.03615995004200030009x>.

6. **Line 138:** The text indicates that moisture contents of "FC, 0.75FC, 0.5FC and 0.25FC were implied". The correct scientific terminology here should be "applied" or "maintained" rather than "implied".

**Authors Response:** The term “implied” has been corrected with “maintained” and can be found in line no 151 in revised marked-up manuscript.

7. **Lines 141–142:** Explain the rationale behind selecting 0.05 M Ca(NO_3_)_2_ as the extracting agent for time-dependent kinetics. While it effectively targets exchangeable fractions without shifting the matrix excessively, comparison with traditional DTPA or water-soluble pools should be justified to demonstrate its physiological relevance to plant uptake.

**Authors Response:** A 0.05 M Ca(NO₃)₂ solution was selected because it is a neutral electrolyte that extracts the readily soluble and readily exchangeable iron pool while minimizing disturbance of the soil matrix and also to make the background solution more uniform with respect to the most exchangeable cation in soil Ca^2+^. Compared with strong chelating extractants such as DTPA, Ca(NO₃)₂ better represents the immediately labile Fe pool that contributes to soil solution chemistry and short-term plant availability, making it suitable for kinetic investigations to record even minute fluctuation in the afore mentioned pools.

8. **Line 153:** The text mentions that the "Debye-Huckel activity correction" was used. Given that soil solution extracts can vary in ionic strength, specify whether the *Extended Debye-Hückel* or the *Davies equation* was automatically prioritized by Visual MINTEQ based on the calculated ionic strength of the extract matrix.

**Authors Response:** The extended Debye-Huckel equation was automatically prioritized by Visual MINTEQ based on the calculated ionic strength of the extract matrix.

9. **Line 168 (Kinetic Models Table):** In the First-order equation and Second-order equation, the minus sign indicates a *desorption/decay* processes where solution concentration decreases over time. However, the data shows that extractable Fe increases over incubation time (from 4 DAI to 45 DAI). Linear forms of accumulation/release equations should feature a positive sign for the rate constants if q_t represents cumulative iron released into the solution phase over time. Please rectify the signs to align with your release data.

a. For the Elovich equation clearly define the physical meaning of the constants

(Initial release rate) and (desorption constant) relative to your soil parameters.

**Authors Response:** The equations presented in Kinetic Models Table are correct and they have been adapted from a similar kind of research work on soils of Hongcheon, Gangwon Province, South Korea by Almaroai et al. (2013).

Reference: Almaroai, Y. A., Usman, A. R., Ahmad, M., Kim, K. R., Vithanage, M., & Sik Ok, Y. (2013). Role of chelating agents on release kinetics of metals and their uptake by maize from chromated copper arsenate-contaminated soil. Environmental technology, 34(6), 747-755.

a **Authors Response:** The parameters of the kinetic model equations including Elovich equation have been added in line no 194 to 199 in revised marked-up manuscript.

10. **Line 190:** The phrase "statistical procedure [30]" is missing its specific package name or programmatic reference within the text. Please explicitly state the R package used (e.g., agricolae, lm, etc.).

**Authors Response:** The statistical analysis section has been revised to specify the software and packages used (Line No 221 in revised marked-up manuscript).

11. **Lines 204–205:** There is a mismatch between the text and the table range summary. Line 204 states that DTPA extractable Fe ranged from 4.31 to 58.07 mg kg⁻¹, but the reported range row at the bottom of Table 2 (Line 109) omits the DTPA range entirely, showing only 3.8 – 22 for CEC. Ensure all parameters discussed in the text are fully summarized within the table boundaries.

**Authors Response:** The Line 235-236 in revised manuscript states that DTPA extractable Fe ranged from 4.31 to 58.07 mg kg⁻¹ is supported by figure 1 where the graphical representation of the DTPA extractable Fe content of the experimental soils are presented along with initial 0.05 M Ca(NO_3_)_2_ extractable iron content.

**Reviewer 2:**

1. The authors rely on Flame Atomic Absorption Spectroscopy (FAAS) and Inductively Coupled Plasma Mass Spectrometry (ICP-MS) to feed their Visual MINTEQ speciation simulations. Crucially, FAAS and ICP-MS yield total element concentrations rather than individual chemical species. While the authors use these total iron concentrations as inputs for Visual MINTEQ to estimate geochemical speciation, it is vital to note that determining true analytical speciation experimentally via these spectroscopic techniques requires a physical separation step (e.g., liquid chromatography) coupled prior to detection. The authors must explicitly clarify this distinction in the text.

**Authors response:** We thank the reviewer for this important observation. The suggestion has been incorporated in the “Geochemical Model” section under “Materials and Methods” section of the manuscript (line no 178 to 187 in revised marked-up manuscript).

2. The manuscript implies or attempts a direct comparison between the simulated species and the total raw experimental data. Could the authors clarify whether it is methodologically feasible to directly validate or compare specific simulated Fe ionic species—such as Fe^2+^, Fe^3+^, or specific organo-mineral complexes—from Visual MINTEQ against empirical data obtained from total FAAS/ICP-MS quantification? If experimental speciation was not performed, the limitations of relying solely on a thermodynamic simulation without analytical validation should be explicitly addressed in the discussion section**.**

**Authors response:** we have revised the Discussion section according to the suggestion to explicitly state that the predicted Fe²⁺, Fe³⁺, hydrolyzed species, and organo-mineral complexes represent equilibrium thermodynamic simulations and should be interpreted as model-based estimates rather than experimentally verified species. We further acknowledge that experimental confirmation of aqueous Fe speciation would require dedicated speciation techniques involving chromatographic separation coupled with elemental detection (e.g., HPLC-ICP-MS or IC-ICP-MS), which were beyond the scope of the present investigation. The revised discussion now explicitly recognizes this limitation and recommends future analytical speciation studies for validating thermodynamic model predictions (line no 478 to 487 in revised marked-up manuscript).

3. The authors collected only bulk surface soil samples. Given that iron dynamics, redox potential, and moisture retention profiles vary dramatically across distinct soil horizons, please justify why deeper soil profiles were not considered or analyzed in this study. Furthermore, please discuss how this surface-centric sampling might limit the broader applicability of your kinetic models.

**Authors response:** The objective of the present study was to investigate iron release kinetics in the agriculturally active plough layer (0–15 cm), which represents the principal root zone for most cultivated crops and is the soil layer routinely used for fertilizer recommendations, soil fertility assessment, and micronutrient diagnosis. we agree that subsoil horizons often differ substantially in redox status, clay mineralogy, organic matter, carbonate content, and moisture dynamics, all of which may influence Fe release mechanisms and accordingly the limitations of the applicability of kinetic models presented in the study, have be added to the discussion section (line no 426-439 in revised marked-up manuscript).

4. I suggest adding the specific peer-reviewed references utilized to carry out the extraction and subsequent quantification of iron (Fe) and aluminum (Al) in the soil extracts.

**Authors response:** The peer reviewed reference has already been added to the manuscript (line no 140 in revised marked-up manuscript), reference number 16.

5. To improve the reproducibility and clarity of the methodology, the authors should briefly describe the chemical rationale or specific advantages of the chosen extraction frameworks used in this work. Specifically, please provide a brief justification for utilizing: DTPA and 0.1 M triethanolamine for Fe extraction; the rapid titration technique for calcium carbonate quantification and; the sodium dithionite–sodium citrate method for sesquioxide extraction.

**Authors response:** The suggestion has been incorporated in the “Characterization of experimental soil samples” segment under “Materials and Methods” segment in the manuscript (line no 131-145 in revised marked-up manuscript).
