# Supplementary material for: Iron release dynamics in Indo-Gangetic alluvial soils under variable moisture conditions: Insights from multi-model kinetic and geochemical speciation analysis
Source: PLoS One. 2026 Aug 28;21(8):e0355846. doi: 10.1371/journal.pone.0355846 (PMC13524240; doi:10.1371/journal.pone.0355846)
Supplement: Peer review history

# Peer review: Decision Letter 0

**DOI:** 10.1371/journal.pone.0355846.r001

**Authors:** Nitin Khandelwal

1 Jul 2026

PLOS One

Dear Dr. Rao,

==============================

**ACADEMIC EDITOR:**

Thank you for submitting your manuscript. Based on the reviewers' evaluations, the study addresses a relevant topic and has merit; however, several important issues related to methodological clarity, data consistency, model interpretation, and presentation must be addressed before further consideration. In particular, the manuscript requires clarification of analytical and modeling approaches, correction of inconsistencies in the text and tables, improved justification of experimental methods, and a more comprehensive discussion of the limitations and implications of the findings.

Please carefully address all reviewer comments provided below and submit a revised manuscript along with a detailed point-by-point response indicating how each concern has been addressed.

==============================

Please submit your revised manuscript by Aug 15 2026 11:59PM. If you will need more time than this to complete your revisions, please reply to this message or contact the journal office at plosone@plos.org. When you're ready to submit your revision, log on to https://www.editorialmanager.com/pone/ and select the 'Submissions Needing Revision' folder to locate your manuscript file.

- A letter that responds to each point raised by the academic editor and reviewer(s). You should upload this letter as a separate file labeled 'Response to Reviewers'.
- A marked-up copy of your manuscript that highlights changes made to the original version. You should upload this as a separate file labeled 'Revised Manuscript with Track Changes'.
- An unmarked version of your revised paper without tracked changes. You should upload this as a separate file labeled 'Manuscript'.

If you would like to make changes to your financial disclosure, please include your updated statement in your cover letter. Guidelines for resubmitting your figure files are available below the reviewer comments at the end of this letter.

If applicable, we recommend that you deposit your laboratory protocols in protocols.io to enhance the reproducibility of your results. Protocols.io assigns your protocol its own identifier (DOI) so that it can be cited independently in the future. For instructions see: https://journals.plos.org/plosone/s/submission-guidelines#loc-laboratory-protocols. Additionally, PLOS ONE offers an option for publishing peer-reviewed Lab Protocol articles, which describe protocols hosted on protocols.io. Read more information on sharing protocols at https://plos.org/protocols?utm_medium=editorial-email&utm_source=authorletters&utm_campaign=protocols.

As the corresponding author, your ORCID iD is verified in the submission system and will appear in the published article. PLOS supports the use of ORCID, and we encourage all coauthors to register for an ORCID iD and use it as well. Please encourage your coauthors to verify their ORCID iD within the submission system before final acceptance, as unverified ORCID iDs will not appear in the published article. *Only*  the individual author can complete the verification step; PLOS staff *cannot*  verify ORCID iDs on behalf of authors.

We look forward to receiving your revised manuscript.

Kind regards,

Nitin Khandelwal

Academic Editor

PLOS One

Journal Requirements:

When submitting your revision, we need you to address these additional requirements.

1. Please ensure that your manuscript meets PLOS ONE's style requirements, including those for file naming. The PLOS ONE style templates can be found at https://journals.plos.org/plosone/s/file?id=wjVg/PLOSOne_formatting_sample_main_body.pdf and https://journals.plos.org/plosone/s/file?id=ba62/PLOSOne_formatting_sample_title_authors_affiliations.pdf

2. Please note that PLOS One has specific guidelines on code sharing for submissions in which author-generated code underpins the findings in the manuscript. In these cases, we expect all author-generated code to be made available without restrictions upon publication of the work. Please review our guidelines at https://journals.plos.org/plosone/s/materials-and-software-sharing#loc-sharing-code and ensure that your code is shared in a way that follows best practice and facilitates reproducibility and reuse.

3. In your Methods section, please provide additional information regarding the permits you obtained for the work. Please ensure you have included the full name of the authority that approved the field site access and, if no permits were required, a brief statement explaining why.

4. Thank you for stating in your Funding Statement:

[Part of the research was supported by a grant from the Bill & Melinda Gates Foundation (Grant 467 number # OPP1215722) sub-grant to India for the Zn mainstreaming project. The funders had no role in study design, data collection and analysis, decision to publish, or preparation of the manuscript.].

Please provide an amended statement that declares *all* the funding or sources of support (whether external or internal to your organization) received during this study, as detailed online in our guide for authors at http://journals.plos.org/plosone/s/submit-now.  Please also include the statement “There was no additional external funding received for this study.” in your updated Funding Statement.

Please include your amended Funding Statement within your cover letter. We will change the online submission form on your behalf.

5. We note that your Data Availability Statement is currently as follows: [All relevant data are within the manuscript and its Supporting Information files.]

Please confirm at this time whether or not your submission contains all raw data required to replicate the results of your study. Authors must share the “minimal data set” for their submission. PLOS defines the minimal data set to consist of the data required to replicate all study findings reported in the article, as well as related metadata and methods (https://journals.plos.org/plosone/s/data-availability#loc-minimal-data-set-definition).

For example, authors should submit the following data:

- The values behind the means, standard deviations and other measures reported;

- The values used to build graphs;

- The points extracted from images for analysis.

Authors do not need to submit their entire data set if only a portion of the data was used in the reported study.

If your submission does not contain these data, please either upload them as Supporting Information files or deposit them to a stable, public repository and provide us with the relevant URLs, DOIs, or accession numbers. For a list of recommended repositories, please see https://journals.plos.org/plosone/s/recommended-repositories.

If there are ethical or legal restrictions on sharing a de-identified data set, please explain them in detail (e.g., data contain potentially sensitive information, data are owned by a third-party organization, etc.) and who has imposed them (e.g., an ethics committee). Please also provide contact information for a data access committee, ethics committee, or other institutional body to which data requests may be sent. If data are owned by a third party, please indicate how others may request data access.

If the reviewer comments include a recommendation to cite specific previously published works, please review and evaluate these publications to determine whether they are relevant and should be cited. There is no requirement to cite these works unless the editor has indicated otherwise.

Additional Editor Comments:

Thank you for submitting your manuscript. Based on the reviewers' evaluations, the study addresses a relevant topic and has merit; however, several important issues related to methodological clarity, data consistency, model interpretation, and presentation must be addressed before further consideration. In particular, the manuscript requires clarification of analytical and modeling approaches, correction of inconsistencies in the text and tables, improved justification of experimental methods, and a more comprehensive discussion of the limitations and implications of the findings.

Please carefully address all reviewer comments provided below and submit a revised manuscript along with a detailed point-by-point response indicating how each concern has been addressed.

[Note: HTML markup is below. Please do not edit.]

Reviewers' comments:

Reviewer's Responses to Questions

**Comments to the Author**

1. Is the manuscript technically sound, and do the data support the conclusions?

Reviewer #1: Yes

Reviewer #2: Partly

**********

2. Has the statistical analysis been performed appropriately and rigorously?

Reviewer #1: Yes

Reviewer #2: Yes

**********

3. Have the authors made all data underlying the findings in their manuscript fully available?

The PLOS Data policy

Reviewer #1: Yes

Reviewer #2: Yes

**********

4. Is the manuscript presented in an intelligible fashion and written in standard English?

Reviewer #1: Yes

Reviewer #2: Yes

**********

Reviewer #1: The temporal release kinetics and geochemical speciation of native Iron (Fe) in Indo-Gangetic Alluvial Soils under varying moisture conditions. The combination of mechanistic kinetic equations with thermodynamic equilibrium modeling (Visual MINTEQ) provides a robust platform to evaluate micronutrient dynamics. However, the paper requires critical modifications to resolve inconsistencies in the text, clarify data parameters, and strengthen the overall scientific validity.

Reviewer #2: I would like to express my gratitude for the invitation to review the manuscript entitled: “Iron Release Dynamics in Indo-Gangetic Alluvial Soils under Variable Moisture Conditions: Insights from Multi-Model Kinetic and Geochemical Speciation Analysis”. The manuscript investigates the kinetics and geochemical speciation of iron release in Indo-Gangetic alluvial soils under shifting moisture regimes. The combination of multi-model kinetic equations with thermodynamic modeling (Visual MINTEQ) addresses a highly relevant topic in soil chemistry and nutrient availability. The study is well-conceived; however, I suggest addressing the following points to strengthen the manuscript:

Section Geochemical Model (Total Concentrations vs. Analytical Speciation):

The authors rely on Flame Atomic Absorption Spectroscopy (FAAS) and Inductively Coupled Plasma Mass Spectrometry (ICP-MS) to feed their Visual MINTEQ speciation simulations. Crucially, FAAS and ICP-MS yield total element concentrations rather than individual chemical species. While the authors use these total iron concentrations as inputs for Visual MINTEQ to estimate geochemical speciation, it is vital to note that determining true analytical speciation experimentally via these spectroscopic techniques requires a physical separation step (e.g., liquid chromatography) coupled prior to detection. The authors must explicitly clarify this distinction in the text.

The manuscript implies or attempts a direct comparison between the simulated species and the total raw experimental data. Could the authors clarify whether it is methodologically feasible to directly validate or compare specific simulated Fe ionic species—such as $Fe^{2+}$, $Fe^{3+}$, or specific organo-mineral complexes—from Visual MINTEQ against empirical data obtained from total FAAS/ICP-MS quantification? If experimental speciation was not performed, the limitations of relying solely on a thermodynamic simulation without analytical validation should be explicitly addressed in the discussion section.

Section Location of Soil Samples:

The authors collected only bulk surface soil samples. Given that iron dynamics, redox potential, and moisture retention profiles vary dramatically across distinct soil horizons, please justify why deeper soil profiles were not considered or analyzed in this study. Furthermore, please discuss how this surface-centric sampling might limit the broader applicability of your kinetic models.

I suggest adding the specific peer-reviewed references utilized to carry out the extraction and subsequent quantification of iron (Fe) and aluminum (Al) in the soil extracts.

To improve the reproducibility and clarity of the methodology, the authors should briefly describe the chemical rationale or specific advantages of the chosen extraction frameworks used in this work. Specifically, please provide a brief justification for utilizing: DTPA and 0.1 M triethanolamine for Fe extraction; the rapid titration technique for calcium carbonate quantification and; the sodium dithionite–sodium citrate method for sesquioxide extraction.

**********

what does this mean?). If published, this will include your full peer review and any attached files.

If you choose “no”, your identity will remain anonymous but your review may still be made public.

**Do you want your identity to be public for this peer review?**  For information about this choice, including consent withdrawal, please see our Privacy Policy

Reviewer #1: **Yes:**  SADASHIV CHATURVEDI

Reviewer #2: No

**********

[NOTE: If reviewer comments were submitted as an attachment file, they will be attached to this email and accessible via the submission site. Please log into your account, locate the manuscript record, and check for the action link "View Attachments". If this link does not appear, there are no attachment files.]

To ensure your figures meet our technical requirements, please review our figure guidelines: https://journals.plos.org/plosone/s/figures

You may also use PLOS’s free figure tool, NAAS, to help you prepare publication quality figures: https://journals.plos.org/plosone/s/figures#loc-tools-for-figure-preparation.

NAAS will assess whether your figures meet our technical requirements by comparing each figure against our figure specifications.

Attachment Submitted filename: geochemical speciation of native Iron_comments.pdf

# Author comment: Author response to Decision Letter 1

**DOI:** 10.1371/journal.pone.0355846.r002

13 Jul 2026

To

Editor-in-Chief

PLOS One

Sub: Revised manuscript entitled “Iron Release Dynamics in Indo-Gangetic Alluvial Soils under Variable Moisture Conditions: Insights from Multi-Model Kinetic and Geochemical Speciation Analysis” [PONE-D-26-16816]

Sir,

Thank you very much for your kind comments/suggestions for improving our manuscript. All the comments/suggestions are incorporated in the revised manuscript. The point-wise response to comments/suggestions is given below:

Authors response to Line-by-Line Specific Comments

Reviewer 1:

1. Lines 27–29: The text states, "...an incubation experiment was carried out with soils of 20 different locations...", but Line 108 in the Materials and Methods section explicitly states, "A total of twenty-five bulk surface (0-15 cm) soil samples were collected...".

This inconsistency must be resolved. Additionally, Table 1 only lists 20 soils (S1 to S20). Please correct the text to reflect the exact number of soils evaluated.

Authors Response: We thank the reviewer for identifying this inconsistency. A total of 20 number of soil samples were collected from 20 different locations of Indo-Gangetic Plain as mentioned in Table 1 and all the 20 soil samples were evaluated. The inconsistency has been rectified in line number 112 in revised marked-up manuscript.

2. Line 74: Clarify what "specific compounds" refers to in this context. Because this paper focuses exclusively on native iron (Fe) release, frame this sentence specifically around iron minerals or desorption behavior to make it contextually relevant.

Authors Response: The sentence has been corrected accordingly to make it contextually relevant (line no 72 to 76 in revised marked-up manuscript).

3. Lines 80–81: The text notes that higher extractability occurs due to the partial reduction of Fe(III) to Fe(II). Because Ca(NO3)2 is an unbuffered salt extractant used to evaluate the water-soluble/exchangeable pool, please state whether any precautions (such as purging with inert gas or immediate acidification) were taken during filtration to prevent the rapid oxidation and precipitation of Fe2+ back into Fe3+ oxyhydroxides.

Authors Response: Thank you for this important observation. The extraction procedure was carried out in closed polypropylene bottles the caps were opened only for addition of the extracting reagent and recapped immediately without prolonged exposure to air and then shaking was done using in environmental shaker. No inert gas purging was employed because the objective of the study was to quantify Ca(NO₃)₂-extractable Fe rather than to determine Fe²⁺ and Fe³⁺ individually. This clarification has now been incorporated into the Materials and Methods section (Line No 155-158 in revised marked-up manuscript).

4. Lines 109–110: The phrase "...covering 5 states and 1 union territory..." should be explicitly verified against the contents of Table 1. Table 1 lists locations from Delhi, Bihar, Haryana, Punjab, and West Bengal, which comprises 4 states and 1 Union Territory (Delhi). Please correct the tally of states.

Authors Response: The 20 soil samples were collected from 5 states and 1 union territory is correct. The 5 states are Bihar (S2, S6 and S10), Haryana (S3, S5, S8 and S9), Punjab (S4 and S13), Uttar Pradesh (S7, S12, S15 and S17) and West Bengal (S11, S14 and S16) and 1 union territory is Delhi (S1, S18, S19 and S20).

5. Lines 126–128: It is mentioned that micronutrient availability was evaluated using

"0.005 M DTPA, 0.01 M CaCl_2, and 0.1 M triethanolamine (TEA)". This standard

Lindsay and Norvell extractant mixture uses 0.005 M DTPA, 0.05 M CaCl2, and 0.1 M TEA. Please double-check if the concentration of CaCl2 was indeed 0.01 M or if this is a typographical error.

Authors Response: We have used the composition of the DTPA extractant as per the research article published by Lindsay and Norvell (1978) where the concentration of CaCl2 is 0.01 M.

Reference: Lindsay, W. L., & Norvell, W. (1978). Development of a DTPA soil test for zinc, iron, manganese, and copper. Soil science society of America journal, 42(3), 421-428. https://doi.org/10.2136/sssaj1978.03615995004200030009x.

6. Line 138: The text indicates that moisture contents of "FC, 0.75FC, 0.5FC and 0.25FC were implied". The correct scientific terminology here should be "applied" or "maintained" rather than "implied".

Authors Response: The term “implied” has been corrected with “maintained” and can be found in line no 151 in revised marked-up manuscript.

7. Lines 141–142: Explain the rationale behind selecting 0.05 M Ca(NO3)2 as the extracting agent for time-dependent kinetics. While it effectively targets exchangeable fractions without shifting the matrix excessively, comparison with traditional DTPA or water-soluble pools should be justified to demonstrate its physiological relevance to plant uptake.

Authors Response: A 0.05 M Ca(NO₃)₂ solution was selected because it is a neutral electrolyte that extracts the readily soluble and readily exchangeable iron pool while minimizing disturbance of the soil matrix and also to make the background solution more uniform with respect to the most exchangeable cation in soil Ca2+. Compared with strong chelating extractants such as DTPA, Ca(NO₃)₂ better represents the immediately labile Fe pool that contributes to soil solution chemistry and short-term plant availability, making it suitable for kinetic investigations to record even minute fluctuation in the afore mentioned pools.

8. Line 153: The text mentions that the "Debye-Huckel activity correction" was used. Given that soil solution extracts can vary in ionic strength, specify whether the Extended Debye-Hückel or the Davies equation was automatically prioritized by Visual MINTEQ based on the calculated ionic strength of the extract matrix.

Authors Response: The extended Debye-Huckel equation was automatically prioritized by Visual MINTEQ based on the calculated ionic strength of the extract matrix.

9. Line 168 (Kinetic Models Table): In the First-order equation and Second-order equation, the minus sign indicates a desorption/decay processes where solution concentration decreases over time. However, the data shows that extractable Fe increases over incubation time (from 4 DAI to 45 DAI). Linear forms of accumulation/release equations should feature a positive sign for the rate constants if q_t represents cumulative iron released into the solution phase over time. Please rectify the signs to align with your release data.

a. For the Elovich equation clearly define the physical meaning of the constants

(Initial release rate) and (desorption constant) relative to your soil parameters.

Authors Response: The equations presented in Kinetic Models Table are correct and they have been adapted from a similar kind of research work on soils of Hongcheon, Gangwon Province, South Korea by Almaroai et al. (2013).

Reference: Almaroai, Y. A., Usman, A. R., Ahmad, M., Kim, K. R., Vithanage, M., & Sik Ok, Y. (2013). Role of chelating agents on release kinetics of metals and their uptake by maize from chromated copper arsenate-contaminated soil. Environmental technology, 34(6), 747-755.

a Authors Response: The parameters of the kinetic model equations including Elovich equation have been added in line no 194 to 199 in revised marked-up manuscript.

10. Line 190: The phrase "statistical procedure [30]" is missing its specific package name or programmatic reference within the text. Please explicitly state the R package used (e.g., agricolae, lm, etc.).

Authors Response: The statistical analysis section has been revised to specify the software and packages used (Line No 221 in revised marked-up manuscript).

11. Lines 204–205: There is a mismatch between the text and the table range summary. Line 204 states that DTPA extractable Fe ranged from 4.31 to 58.07 mg kg⁻¹, but the reported range row at the bottom of Table 2 (Line 109) omits the DTPA range entirely, showing only 3.8 – 22 for CEC. Ensure all parameters discussed in the text are fully summarized within the table boundaries.

Authors Response: The Line 235-236 in revised manuscript states that DTPA extractable Fe ranged from 4.31 to 58.07 mg kg⁻¹ is supported by figure 1 where the graphical representation of the DTPA extractable Fe content of the experimental soils are presented along with initial 0.05 M Ca(NO3)2 extractable iron content.

Reviewer 2:

1. The authors rely on Flame Atomic Absorption Spectroscopy (FAAS) and Inductively Coupled Plasma Mass Spectrometry (ICP-MS) to feed their Visual MINTEQ speciation simulations. Crucially, FAAS and ICP-MS yield total element concentrations rather than individual chemical species. While the authors use these total iron concentrations as inputs for Visual MINTEQ to estimate geochemical speciation, it is vital to note that determining true analytical speciation experimentally via these spectroscopic techniques requires a physical separation step (e.g., liquid chromatography) coupled prior to detection. The authors must explicitly clarify this distinction in the text.

Authors response: We thank the reviewer for this important observation. The suggestion has been incorporated in the “Geochemical Model” section under “Materials and Methods” section of the manuscript (line no 178 to 187 in revised marked-up manuscript).

2. The manuscript implies or attempts a direct comparison between the simulated species and the total raw experimental data. Could the authors clarify whether it is methodologically feasible to directly validate or compare specific simulated Fe ionic species—such as Fe2+, Fe3+, or specific organo-mineral complexes—from Visual MINTEQ against empirical data obtained from total FAAS/ICP-MS quantification? If experimental speciation was not performed, the limitations of relying solely on a thermodynamic simulation without analytical validation should be explicitly addressed in the discussion section.

Authors response: we have revised the Discussion section according to the suggestion to explicitly state that the predicted Fe²⁺, Fe³⁺, hydrolyzed species, and organo-mineral complexes represent equilibrium thermodynamic simulations and should be interpreted as model-based estimates rather than experimentally verified species. We further acknowledge that experimental confirmation of aqueous Fe speciation would require dedicated speciation techniques involving chromatographic separation coupled with elemental detection (e.g., HPLC-ICP-MS or IC-ICP-MS), which were beyond the scope of the present investigation. The revised discussion now explicitly recognizes this limitation and recommends future analytical speciation studies for validating thermodynamic model predictions (line no 478 to 487 in revised marked-up manuscript).

3. The authors collected only bulk surface soil samples. Given that iron dynamics, redox potential, and moisture retention profiles vary dramatically across distinct soil horizons, please justify why deeper soil profiles were not considered or analyzed in this study. Furthermore, please discuss how this surface-centric sampling might limit the broader applicability of your kinetic models.

Authors response: The objective of the present study was to investigate iron release kinetics in the agriculturally active plough layer (0–15 cm), which represents the principal root zone for most cultivated crops and is the soil layer routinely used for fertilizer recommendations, soil fertility assessment, and micronutrient diagnosis. we agree that subsoil horizons often differ substantially in redox status, clay mineralogy, organic matter, carbonate content, and moisture dynamics, all of which may influence Fe release mechanisms and accordingly the limitations of the applicability of kinetic models presented in the study, have be added to the discussion section (line no 426-439 in revised marked-up manuscript).

4. I suggest adding the specific peer-reviewed references utilized to carry out the extraction and subsequent quantification of iron (Fe) and aluminum (Al) in the soil extracts.

Authors response: The peer reviewed reference has already been added to the manuscript (line no 140 in revised marked-up manuscript), reference number 16.

5. To improve the reproducibility and clarity of the methodology, the authors should briefly describe the chemical rationale or specific advantages of the chosen extraction frameworks used in this work. Specifically, please provide a brief justification for utilizing: DTPA and 0.1 M triethanolamine for Fe extraction; the rapid titration technique for calcium carbonate quantification and; the sodium dithionite–sodium citrate method for sesquioxide extraction.

Authors response: The suggestion has been incorporated in the “Characterization of experimental soil samples” segment under “Materials and Methods” segment in the manuscript (line no 131-145 in revised marked-up manuscript).

Attachment Submitted filename: Authors_response.docx

# Peer review: Decision Letter 1

**DOI:** 10.1371/journal.pone.0355846.r003

**Authors:** Nitin Khandelwal

27 Jul 2026

Iron Release Dynamics in Indo-Gangetic Alluvial Soils under Variable Moisture Conditions: Insights from Multi-Model Kinetic and Geochemical Speciation Analysis

PONE-D-26-16816R1

Dear Dr. Rao,

We’re pleased to inform you that your manuscript has been judged scientifically suitable for publication and will be formally accepted for publication once it meets all outstanding technical requirements.

Within one week, you’ll receive an e-mail detailing the required amendments. When these have been addressed, you’ll receive a formal acceptance letter and your manuscript will be scheduled for publication.

An invoice will be generated when your article is formally accepted. Please note, if your institution has a publishing partnership with PLOS and your article meets the relevant criteria, all or part of your publication costs will be covered. Please make sure your user information is up-to-date by logging into Editorial Manager at Editorial Manager® and clicking the ‘Update My Information' link at the top of the page. For questions related to billing, please contact billing support.

If your institution or institutions have a press office, please notify them about your upcoming paper to help maximize its impact. If they’ll be preparing press materials, please inform our press team as soon as possible -- no later than 48 hours after receiving the formal acceptance. Your manuscript will remain under strict press embargo until 2 pm Eastern Time on the date of publication. For more information, please contact onepress@plos.org.

Kind regards,

Nitin Khandelwal

Academic Editor

PLOS One

Additional Editor Comments (optional):

Reviewers' comments:

Reviewer's Responses to Questions

**Comments to the Author**

Reviewer #1: All comments have been addressed

**********

2. Is the manuscript technically sound, and do the data support the conclusions?

Reviewer #1: (No Response)

**********

3. Has the statistical analysis been performed appropriately and rigorously?

Reviewer #1: (No Response)

**********

4. Have the authors made all data underlying the findings in their manuscript fully available?

The PLOS Data policy

Reviewer #1: (No Response)

**********

5. Is the manuscript presented in an intelligible fashion and written in standard English?

Reviewer #1: (No Response)

**********

Reviewer #1: Accepted in the current form, all comments have been addressed.

Accepted in the current form, all comments have been addressed.

**********

what does this mean?). If published, this will include your full peer review and any attached files.

If you choose “no”, your identity will remain anonymous but your review may still be made public.

**Do you want your identity to be public for this peer review?**  For information about this choice, including consent withdrawal, please see our Privacy Policy

Reviewer #1: **Yes:**  Sadashiv Chaturvedi

**********

# Editor report: Acceptance letter

**DOI:** 10.1371/journal.pone.0355846.r004

**Authors:** Nitin Khandelwal

PONE-D-26-16816R1

PLOS One

Dear Dr. Rao,

I'm pleased to inform you that your manuscript has been deemed suitable for publication in PLOS One. Congratulations! Your manuscript is now being handed over to our production team.

At this stage, our production department will prepare your paper for publication. This includes ensuring the following:

* All references, tables, and figures are properly cited

* All relevant supporting information is included in the manuscript submission,

* There are no issues that prevent the paper from being properly typeset

You will receive further instructions from the production team, including instructions on how to review your proof when it is ready. Please keep in mind that we are working through a large volume of accepted articles, so please give us a few days to review your paper and let you know the next and final steps.

Lastly, if your institution or institutions have a press office, please let them know about your upcoming paper now to help maximize its impact. If they'll be preparing press materials, please inform our press team within the next 48 hours. Your manuscript will remain under strict press embargo until 2 pm Eastern Time on the date of publication. For more information, please contact onepress@plos.org.

You will receive an invoice from PLOS for your publication fee after your manuscript has reached the completed accept phase. If you receive an email requesting payment before acceptance or for any other service, this may be a phishing scheme. Learn how to identify phishing emails and protect your accounts at https://explore.plos.org/phishing.

If we can help with anything else, please email us at customercare@plos.org.

Thank you for submitting your work to PLOS One and supporting open access.

Kind regards,

PLOS ONE Editorial Office Staff

on behalf of

Dr. Nitin Khandelwal

Academic Editor

PLOS One
